# A Dual‐Kinetic Control Strategy for Designing Nano‐Metamaterials: Novel Class of Metamaterials with Both Characteristic and Whole Sizes of Nanoscale

**DOI:** 10.1002/advs.202205595

**Published:** 2022-11-15

**Authors:** Guanhua Xu, Mengmeng Li, Qiyue Wang, Feng Feng, Qi Lou, Yi Hou, Junfeng Hui, Peisen Zhang, Li Wang, Li Yao, Shijie Qin, Xiaoping Ouyang, Dazhuan Wu, Daishun Ling, Xiuyu Wang

**Affiliations:** ^1^ Institute of Process Equipment College of Energy Engineering Zhejiang University Hangzhou 310027 P. R. China; ^2^ Frontiers Science Center for Transformative Molecules School of Chemistry and Chemical Engineering National Center for Translational Medicine Shanghai Jiao Tong University Shanghai 200240 P. R. China; ^3^ College of Life Science and Technology Beijing University of Chemical Technology Beijing 100029 P. R. China; ^4^ Shaanxi Key Laboratory of Degradable Biomedical Materials School of Chemical Engineering Northwest University Xi'an Shaanxi 710069 P. R. China; ^5^ Beijing National Laboratory for Molecular Sciences State Key Laboratory for Structural Chemistry of Unstable and Stable Species Institute of Chemistry Chinese Academy of Science Beijing 100190 P. R. China; ^6^ School of Chemistry and Chemical Engineering University of Chinese Academy of Science Beijing 100049 P. R. China

**Keywords:** block copolymer self‐assembly, hierarchical structure, kinetic control, nano‐metamaterials, T1‐weighted magnetic resonance imaging

## Abstract

Increasingly intricate in their multilevel multiscale microarchitecture, metamaterials with unique physical properties are challenging the inherent constraints of natural materials. Their applicability in the nanomedicine field still suffers because nanomedicine requires a maximum size of tens to hundreds of nanometers; however, this size scale has not been achieved in metamaterials. Therefore, “nano‐metamaterials,” a novel class of metamaterials, are introduced, which are rationally designed materials with multilevel microarchitectures and both characteristic sizes and whole sizes at the nanoscale, investing in themselves remarkably unique and significantly enhanced material properties as compared with conventional nanomaterials. Microarchitectural regulation through conventional thermodynamic strategy is limited since the thermodynamic process relies on the frequency‐dependent effective temperature, *T*_eff_ *(ω)*, which limits the architectural regulation freedom degree. Here, a novel dual‐kinetic control strategy is designed to fabricate nano‐metamaterials by freezing a high‐free energy state in a *T*_eff_ *(ω)*‐constant system, where two independent dynamic processes, non‐solvent induced block copolymer (BCP) self‐assembly and osmotically driven self‐emulsification, are regulated simultaneously. Fe^3+^‐“onion‐like core@porous corona” (Fe^3+^‐OCPCs) nanoparticles (the products) have not only architectural complexity, porous corona and an onion‐like core but also compositional complexity, Fe^3+^ chelating BCP assemblies. Furthermore, by using Fe^3+^‐OCPCs as a model material, a microstructure‐biological performance relationship is manifested in nano‐metamaterials.

## Graphical abstract

“Nano‐metamaterials,” a novel class of metamaterials, are introduced, which are rationally designed materials with multilevel microarchitectures and both characteristic sizes and whole sizes at the nanoscale, investing in themselves remarkably unique material properties as compared with conventional nanomaterials. A dual‐kinetic control strategy to fabricate nano‐metamaterials is presented, regulating osmotically driven self‐emulsification and nonsolvent‐induced block copolymer self‐assembly simultaneously.

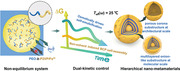

## 1 Introduction

Metamaterials are carefully structured hierarchical materials with multilevel ordered microarchitectures, whose unconventional effective physical properties arose from both the rationally designed artificial structural units (typically periodic) and the careful control over their key parameters.^[^ ^1^ ^]^ Compared with traditional materials with single‐level microarchitecture, metamaterials exhibit multiple levels of complexity, which can be classified according to 1) architecture (e.g., manipulating geometric arrangements), 2) composition (e.g., conjugating different components), and 3) the integration of both architectural and compositional diversity into one superstructure.^[^ ^2^ ^]^ This feature endows metamaterials with properties and functionalities that differ from and surpass those which simply add up the constituent materials.

**Scheme 1:**
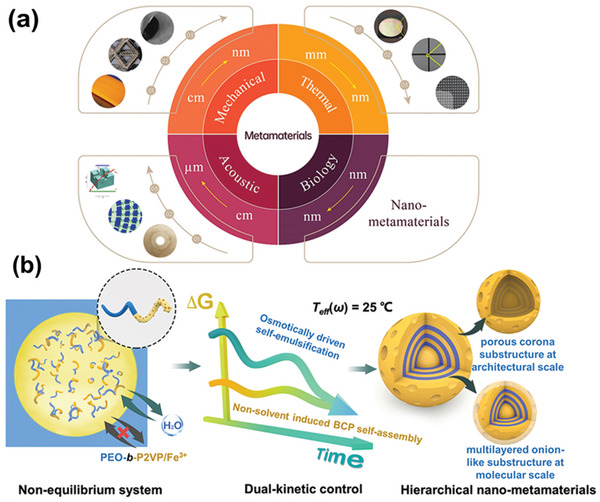
a) The classification of metamaterials based on their scalability. Scheme of mechanical metamaterials. Adapted with permission.^[39^ ^]^ Copyright 2016, Macmillan Publishers Limited. Scheme of acoustic metamaterials. Adapted with permission.^[40^ ^]^ Copyright 2011, The American Physical Society. Scheme of thermal metamaterials. Adapted with permission.^[41^ ^]^ Copyright 2019, Wiley‐VCH. b) Schematic illustration showing the fabrication and physical appearance of the Fe^3+^‐OCPCs based on the proposed dual‐kinetic control strategy. Right: the yellow region represents P2VP/Fe^3+^ and the blue region represents PEO.

In the past two decades, metamaterials that disrupt thermal, acoustic, and mechanical fields and that have highly unusual properties, such as wide‐range thermal expansion coefficients, stimuli triggered negative constitutive parameters, and reprogrammable stiffness or dissipation, have been demonstrated (**Scheme** **1**a).^[^ ^3^ ^]^ Though significant progress has been made in the research of metamaterials, their applicability in certain areas still suffers because of their resistance to size tailoring. For example, despite the efforts that have been demonstrated successful in producing metamaterials with a minimum size of nanometers and maximum size of centimeters or even meters, the applicability of metamaterials falls short in nanomedicine fields which requires a maximum size scale of tens to hundreds of nanometers. Here, we produced a series of multilevel multiscale metamaterials, which not only consisted of controllable ordered multilevel multiscale microarchitecture but also had scalability that is consistent with nanomedicine application requirements. Such metamaterials were defined as “nano‐metamaterials:” a novel class of metamaterials that had a maximum size scale at nanometers. As we know, the development of hierarchical nano‐metamaterials with controllable architectural and compositional complexity is significantly challenging, mainly because the creation of a simple structure conforms to the law of minimum energy.^[^ ^4^ ^]^

According to the physicist Richard Feynman, when a whole system approaches minimum energy, this system is in thermodynamic equilibrium.^[^ ^5^ ^]^ For a system in thermodynamic equilibrium, *T*_eff_ *(w)*, a frequency‐dependent effective temperature, is constant. Deviation from the *T*_eff_ *(w)* triggers the system out of thermodynamic equilibrium, leading to a new equilibrium system with a new structure.^[^ ^6^ ^]^ Despite the theoretical feasibility, exploiting the thermodynamic process as a method to regulate structures suffers from several limitations. First, bioactive material has a constant *T*_eff_ *(w)* that is equal to the temperature of its surroundings and is at thermodynamic equilibrium. Pronounced deviations of *T*_eff_ *(w)* inactive the living materials such as proteins, membranes, and organelles.^[^ ^7^ ^]^ Second, despite the theoretical feasibility, the thermodynamic process relies only on the *T*_eff_ *(w)*, which limits the architectural regulation (equilibrium structures) freedom degrees. Therefore, it is difficult to construct hierarchical nano‐metamaterials from the point of thermodynamics.

Compared with the thermodynamic equilibrium state that relies solely on the *T*_eff_ *(w)*, the dynamic pathway is a time‐dependent manner, which is all about the process.^[^ ^8^ ^]^ It means that a many‐dimensional parameter space of processing variables provides opportunities for creating a wide diversity of non‐equilibrium structures in a *T*_eff_ *(w)*‐constant system.^[^ ^9^ ^]^ As a conceptual model for regulating dynamic processes, we developed a novel dual‐kinetic control strategy, in which two kinetic pathways, block copolymers (BCP) self‐assembly and droplet self‐emulsifying, were controlled simultaneously (Scheme 1b). On the basis of manipulating two different kinetic processes, an unusual dual‐phase separation occurred, including nonsolvent‐induced microphase separation and osmotically driven macrophase separation.^[^ ^10^ ^]^ This elegant approach allowed the program of droplet self‐emulsifying to synchronize with BCP self‐assembly, yielding multilevel multiscale nano‐metamaterials including multilayered onion‐like core substructure at the molecular scale and porous corona substructure at the architectural scale. In addition, the nonergodicity of BCP self‐assembly provided the opportunity to further encapsulate and control the spatial distribution of functional molecules or nanoparticles in the assemblies, leading to the architectures with controllable architectural and compositional complexity under time‐dependent kinetic control.^[^ ^8^, ^11^ ^]^ Utilizing the method, the multilevel multiscale Fe^3+^‐“onion‐like core@porous corona” nanoparticles (Fe^3+^‐OCPCs) were prepared, which comprised two substructures: i) an onion‐like core; and ii) a hierarchically porous corona. Such hierarchical nanoparticles were termed as “nano‐metamaterials.” We defined that nano‐metamaterials were rationally designed materials with multilevel multiscale microarchitectures and both characteristic sizes and whole sizes at the nanoscale, investing in themselves remarkably unique and significantly enhanced material properties, such as optical, ferroelectric, and biological properties, as compared with conventional nanoparticles. Furthermore, by using this well‐defined “nano‐metamaterials” as a model material, a microstructure‐biological performance relationship was manifested in nano‐metamaterials. The simulated and experimental results demonstrated that compared to conventional homogeneous nanoparticles, the confinement of Fe^3+^ ions in the hierarchical microarchitecture of Fe^3+^‐OCPCs changes two important factors, the residence time (*τ*_m_) and the characteristic tumbling time (*τ*_R_), thus enhancing *r*_1_ relaxivity.

## 2 Results and Discussion

**Figure 1:**
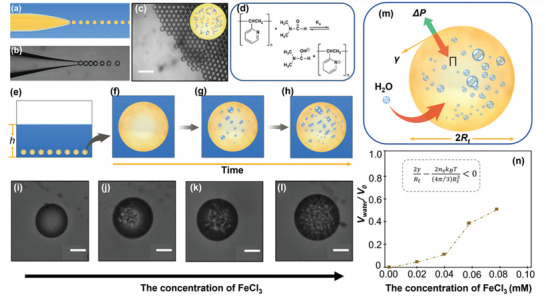
Self‐emulsification process in the monodispersed droplets system. a). Schematic illustration of a microfluidic device. b) Formation of homogeneous oil‐in‐water emulsions using a microfluidic device. c) Monodisperse homogeneous emulsion droplets of DMF + CH_2_Cl_2_ mixture containing PEO‐*b*‐P2VP/Fe^3+^ after collection in a container. The scale bar is 100 µm. d) Proposed mechanism for the deprotonation of P2VP in basic DMF + CH_2_Cl_2_ mixture. e–g) Schematic illustration of the evolution of droplets during the self‐emulsification process: from a single homogeneous droplet to the heterogeneous multi‐emulsions. i–l) Optical microscopy images of the structure of emulsion droplets with different Fe^3+^ concentration, i) *C*_Fe_^3+^ = 0 mm, j) *C*_Fe_^3+^ = 0.02 mm, k) *C*_Fe_^3+^ = 0.04 mm, and l) *C*_Fe_^3+^ = 0.06 mm, respectively. Arrow indicates the direction of increasing concentration of Fe^3+^ in the initial emulsion droplets. The scale bar is 10 µm. m) Schematic of the proposed mechanism for the self‐emulsification process. n) The volume ratio of secondary droplets in the initial emulsion droplets as a function of Fe^3+^ concentration. Increasing Fe^3+^ concentration provides higher osmotic pressure that counterbalances the Laplace pressure, drawing more surrounding water into the emulsion droplets, and finally intensifying the self‐emulsification process.

BCP are generally two or more thermodynamically incompatible homopolymer chains linked together through covalent bonds.^[^ ^12^ ^]^ BCP assemblies, known as “typical nonequilibrium systems,” can be programmed via rational design of arbitrary dynamic pathways toward a global free‐energy minimum.^[^ ^13^ ^]^ It means that controlling the dynamic process of BCP self‐assembly provides new levels of tailorability to nanoscale structures. Previous studies have proved that solvent quality is key to regulating the dynamic pathway of BCP self‐assembly.^[^ ^14^ ^]^ Therefore, over the past 2 decades, basic considerations of how solvent quality affects the dynamic pathway of the BCP self‐assembly are always present.^[^ ^8^ ^]^ Instead, different from the classical consideration, our communication established a kinetic control strategy that had non‐solvent diffusion programmed. This strategy was based on a unique semipermeable droplet system, where two mutually independent kinetic processes occurred, including nonsolvent‐induced BCP self‐assembly and osmotically driven self‐emulsification. By controlling these two processes, we obtained architecturally and compositionally controllable complex nanoparticles, which consisted of two substructures: a multilayered onion‐like core substructure at the molecular scale and a porous corona substructure at the architectural scale (Scheme 1b). To construct the semipermeable confined space, we produced monodisperse and micrometer‐sized droplets using a microfluidic device (as shown in **Figure** **1**a,b), which was a powerful technique to produce calibrated emulsions.^[^ ^15^ ^]^ As displayed in Figure 1a, the dispersed oil phase (O, yellow region) was composed of diluted Fe^3+^ and poly(ethylene oxide)‐block‐poly(2‐vinylpyridine) (PEO‐*b*‐P2VP) (typical BCP) in DMF + CH_2_Cl_2_ solution (volume ratio = 1:10). The continuous water phase (W, blue region) comprised 0.4 mg mL^−1^ poly(vinyl alcohol) (PVA). The dispersed phase flowed through the exit orifice and ruptured to form monodisperse emulsion droplets inside the orifice as a result of interfacial tension.^[^ ^10^ ^]^ Compared to traditional hard confined spaces, such as SiO_2_ hollow colloids, the emulsion droplets were intrinsically out of thermodynamic equilibrium. The semipermeable nature of the droplet interfaces allowed for programming the solvent diffusion while maintaining the BCP necessary for self‐assembly.^[^ ^16^ ^]^

**Figure 2:**
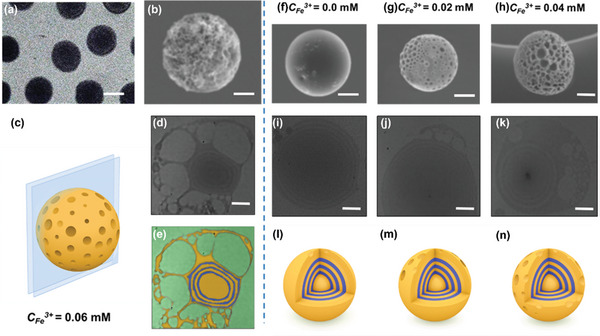
Characterization of the hierarchical structure of Fe^3+^‐OCPCs. a) Transmission electron microscopy (TEM) image of monodisperse Fe^3+^_0.06_‐OCPCs. The scale bar is 500 nm b) Scanning electron microscope (SEM) image showing the wrinkled and porous surface of Fe^3+^_0.06_‐OCPCs, the scale bar is 100 nm. c) Schematic illustration of Fe^3+^_0.06_‐OCPCs sliced in different angles. d) High‐resolution TEM (HR‐TEM) image of the ultra‐thin cross section of Fe^3+^_0.06_‐OCPCs, the scale bar is 50 nm. e) HR‐TEM images of Fe^3+^_0.06_‐OCPCs slices with pseudo color on. The blue color indicates PEO blocks and the yellow color indicates P2VP/Fe^3+^ blocks. f–h) SEM images of the Fe^3+^‐OCPCs produced from different Fe^3+^ concentrations of 0, 0.02, and 0.04 mm, respectively. The scale bar is 100 nm. i–k) HR‐TEM images of the ultra‐thin cross section of Fe^3+^‐OCPCs produced different Fe^3+^ concentrations of 0, 0.02, and 0.04 mm, respectively. The scale bar is 50 nm. l–n) Schematic illustration of Fe^3+^‐OCPCs produced from emulsion droplets containing different Fe^3+^ concentrations of 0, 0.02, and 0.04 mm.

Based on this novel confined space, we established a time‐dependent interaction system via cascading two subsystems: i) a multi‐emulsion droplets subsystem that was initiated by osmotic pressure; ii) a self‐assembly BCP subsystem that was driven by a nonsolvent action. As a proof of concept, PEO‐*b*‐P2VP was selected as a model molecule for several reasons. 1) PEO‐*b*‐P2VP as a typical BCP displays multiple self‐assembly behaviors in solution.^[^ ^17^ ^]^ 2) PEO‐*b*‐P2VP is biocompatible and has been widely explored for biomedical applications such as drug delivery and cancer therapy.^[^ ^18^ ^]^ 3) P2VP shows metal ion affinity and could introduce desired properties of the metal ions into the hierarchical microarchitecture such as catalytic activity, chemical sensing behavior, or spectroscopic response.^[^ ^19^ ^]^ On the other hand, owing to the complex and multi‐fold functions of Fe^3+^, we selected paramagnetic Fe^3+^ ions as the chelates.^[^ ^20^ ^]^ It has been generally recognized that the P2VP is a weak base in acidic solutions due to the presence of the unprotonated N on the pyridine ring. However, the *α*‐H of P2VP also provides protons because the special resonance structures of the pyridine rings stabilized the deprotonated structures.^[^ ^21^ ^]^ In a basic solvent, the relative basicity of the N decreases, and the relative acidity of the *α*‐H increases (Figure 1d). Therefore, in a basic DMF + CH_2_Cl_2_ mixture (i.e., pH_DMF_ = 11.3), amphiprotic P2VP released its *α*‐H to form anionic blocks(P2VP^−^) and was subsequently neutralized by Fe^3+^, forming PEO‐*b*‐P2VP/Fe^3+^ complex. This chelation was further demonstrated by the Fourier transform infrared (FT‐IR) absorption spectrum (Figure S1, Supporting Information), in which the peaks corresponding to pyridine rings shifted to higher frequencies due to the coordinative bonds between N and Fe^3+^. As shown in Figure 1c, the emulsion droplets (28 µm) consisting of the PEO‐*b*‐P2VP/Fe^3+^ complex appeared as a homogeneous single phase at the beginning. However, the droplets no longer kept their homogeneity later on but instead underwent a self‐emulsification process, in which tiny secondary water droplets of 1–4 µm formed spontaneously, due to the diffusion of water molecules into the emulsion droplets through the semipermeable oil/water interface.^[^ ^10^, ^15^ ^]^ Figure 1e–h, the formation of tiny secondary droplets within the primary droplets completed within 30 min. Notably, as the secondary droplets were less dense than the surrounding organic solvent, they floated upward, which caused the formation of the porous corona substructure in the final product shown in **Figure** **2**d.^[^ ^22^ ^]^ Heterogeneous multi‐emulsion droplets, as shown in the sequential stages of self‐emulsification (Figure 1f–h), occurred from the combined action of the spontaneous transport of water molecules and the subsequent macrophase separation in the primary emulsions.

Indeed, the spontaneous transport of water from the bulk continuous phase into micrometer‐sized droplets, which were experiencing a Laplace pressure of about 10 kPa owing to the solvent/water interfacial tension, seemed at first to contradict basic thermodynamic principles.^[^ ^22^ ^]^ However, Fe^3+^ in our system served not only as chelates but also as osmolytes, providing an osmotic driving force (Equation (1)) to counterbalance the Laplace pressure (Equation (2)), thus promoting water transport into the homogeneous droplets and formation of multi‐emulsion droplets (Figure 1m).^[^ ^22^ ^]^

Here, we assumed that the pressure drop along the radius of the droplets was negligible owing to the relatively small curvature of micrometer‐sized droplets. The Laplace pressure difference (Δ*P*) between the interior and exterior of a droplet with a radius *R*_t_ resulting from the solvent/water interfacial tension (*γ*) is given by

(1) $$\Delta P=\frac{2\gamma }{R_{t}}$$

Since the number of Fe^3+^ kept constant during the osmotic inflation of the droplet. The osmotic pressure *∏* in the droplet containing Fe^3+^ of a number of *n*_s_ ions is given by

(2) $$\prod =\frac{2n_{s}k_{B}T}{\left(4\pi /3\right)R_{t}^{3}}$$

When the osmotic driving force exceeded the Laplace pressure (Equation (3)), self‐emulsification occurred, yielding multi‐emulsion droplets.

(3) $$\Delta P^{\prime }=\frac{2\gamma }{R_{t}}-\frac{2n_{s}k_{B}T}{\left(4\pi /3\right)R_{t}^{3}}<0$$

where *R*_t_ is the radius of the droplets at time *t*; *γ*, the interfacial tension of a droplet; *k*_B_, the Plank constant; and *n*_s_, the number of Fe^3+^ in the primary droplets. Since the temperature during the self‐emulsification process was constant, there exists a direct relationship between *n*_s_ and *∏* (Equation (2)). Consequently, a higher *n*_s_ induced a higher *∏*, leading to a higher *∆P*′ according to Equation (3) for the self‐emulsification process. Therefore, *n*_s_ of Fe^3+^ in the primary droplets provided a means to kinetically regulate the self‐emulsification process by programming water diffusion from the continuous phase to the droplets. To test this possibility, we prepared a primary o/w droplet with a size of about 28 µm that contained the amount of Fe^3+^ varied from 0 to 9 µm. As shown in Figure 1i–l, the more Fe^3+^ ions were introduced into the initial droplets, the higher the osmotic pressure was triggered, thus the more water diffusion (Figure 1n) and the more intense the self‐emulsification process, as indicated by the increased number of the interior secondary tiny droplets. Therefore, modulating the amount of Fe^3+^ provided a means to fine‐tune the self‐emulsification intensity and the characterization of macrophase‐separated droplets.^[^ ^22^ ^]^ Therefore, we developed a single‐step process for the preparation of multi‐emulsion droplets (typically made in a multi‐step process),^[^ ^22^ ^]^ where the structures of the multi‐emulsion droplets were tailored simply by Fe^3+^‐induced osmotic pressure. The new paradigm, based on osmotic pressure‐mediated self‐emulsification and macrophase separation, was envisaged to expand to include other functional cations and active molecules to produce complex droplets, which are highly structured fluids with scientific and commercial value. The high degree of controllability afforded by this method rendered itself a flexible and promising technique.

When macrophase separation occurred in this system, it should be borne in mind that water, the most hydrophilic and polar medium, is frequently used as a nonsolvent to induce the rich self‐assembly behaviors of BCPs by microphase separation.^[^ ^23^ ^]^ Meanwhile, the thermodynamic incompatibility between PEO and P2VP blocks could result in a classic self‐assembly behavior.^[^ ^24^ ^]^ Considering these two factors, the water transport will decrease the solvent quality while increasing the entropic penalty during the macrophase‐separated process.^[^ ^25^ ^]^ When the entrance of water continued beyond the onset of entropic penalty, which was required for initiating the copolymer self‐assembly, PEO‐P2VP self‐assembly was induced in order to decrease the entropic penalty, ultimately locking the PEO‐*b*‐P2VP/Fe^3+^ copolymer complex into one of a wide range of possible assembly morphologies.^[^ ^8^, ^26^ ^]^ As a result, the self‐assembly of the PEO‐*b*‐P2VP/Fe^3+^ complex in the droplets became kinetically trapped, yielding a classic onion‐like core substructure because of microphase separation.^[^ ^27^ ^]^ The onion‐like core substructure caused by this microphase separation, combined with the porous corona substructure resulted from macrophase separation, ultimately formed hierarchical Fe^3+^‐“onion‐like core@porous corona” nanoparticles (defined as Fe^3+^‐OCPCs, Figure 2c–e). Therefore, the formation of the hierarchical Fe^3+^‐OCPCs nanoparticles was a direct outcome of two different kinetic pathways: the nonsolvent‐induced PEO‐*b*‐P2VP/Fe^3+^ self‐assembly pathway, which formed the onion‐like core substructure at the molecular scale, and the osmotically driven self‐emulsification pathway, which formed the porous corona substructure at the architectural scale.

Because the self‐emulsification process was mediated by the number of Fe^3+^, we selected the droplet series with the highest Fe^3+^ (*C*_Fe_^3+^ = 0.06 mm) amount in the beginning and studied their morphological development. As a result, nanoparticles with the highest architectural complexity were obtained after the solidification of primary droplets, defined as Fe^3+^_0.06_‐OCPCs. The elemental composition and the Fe^3+^ concentration of Fe^3+^_0.06_‐OCPCs were determined by Figure S2, Supporting Information, and the ICP data. These Fe^3+^_0.06_‐OCPCs exhibited spherical morphology with an average diameter of 560 ± 36 nm, data drawn from analysis of more than 100 Fe^3+^_0.06_‐OCPCs in transmission electron microscopy (TEM) images (Figure 2a). Additionally, Fe^3+^_0.06_‐OCPCs were monodisperse with a polydispersity index (PDI) of 0.03 (Figure S3, Supporting Information), which was reserved by the microfluidic techniques and could satisfy biomedical applications that require high monodispersity.^[^ ^15^ ^]^ Moreover, because of the intensified self‐emulsification process, these monodisperse nanoparticles exhibited wrinkled and porous surfaces, leading to a higher facet exposure (Figure 2b).

To study the internal structure of Fe^3+^_0.06_‐OCPCs, we sliced Fe^3+^_0.06_‐OCPCs from different angles (Figure 2c). Figure 2d shows the high‐resolution TEM images of the ultra‐thin cross section of Fe^3+^_0.06_‐OCPCs. Because the Fe^3+^ ion has a high atomic number, P2VP/Fe^3+^ domains have higher electron density contrast than that of PEO domains. As a result, we distinguished the P2VP/Fe^3+^and PEO domains of Fe^3+^_0.06_‐OCPCs without staining.^[^ ^28^ ^]^ The multilevel microarchitecture was clear‐cut in Fe^3+^_0.06_‐OCPCs including an onion‐like core and a heterogeneous porous corona. The outermost corona of Fe^3+^_0.06_‐OCPCs was about 50 nm in thickness, where pores on multiple length scales (incorporating mesopores (2 to 50 nm) and macropores (>50 nm)) scattered unevenly. The “onion‐like” core was determined to be 190 ± 10 nm and possessed three alternating copolymer layers, the dark P2VP/Fe^3+^ layer of around 15 nm (orange) and the bright PEO layer (blue) of about 13 nm (Figure 2e). Additionally, the porous corona substructure and the “onion‐like” core substructure were further demonstrated by Brunauer–Emmett–Teller (BET) and small angle X‐ray scattering (SAXS). The pore size distribution profile obtained from BET (Figure S4, Supporting Information) showed three major peaks at 5–7.5, 7.5–22, and 22–61 nm, indicating that besides the intrinsic micropores (<2 nm), our Fe^3+^‐OCPCs contained mesopores (2–50 nm) and macropores (>50 nm), which were also observed from Figure 2d. The SAXS pattern (Figure S5, Supporting Information) of Fe^3+^‐OCPCs (red line) exhibited two scattering peaks compared to homogeneous Fe^3+^‐P2VP nanoparticles (black line), which were attributed to the periodic multilayered onion‐like shells, similar to the work previously reported.^[^ ^29^ ^]^ Taken together, these results confirmed the architectural complexity of Fe^3+^‐OCPCs.

Based on the observation that the number of Fe^3+^ ions greatly influenced the self‐emulsification process, we presumed that the architectural complexity of Fe^3+^‐OCPCs could be further regulated by adjusting the number of Fe^3+^ in the initial droplets. To demonstrate the feasibility of this presumption, we produced a wide spectrum of Fe^3+^‐OCPCs nanoparticles with different Fe^3+^ amounts (*C*_Fe_^3+^ = 0, 0.02, 0.04 mm), which were later defined as Fe^3+^_0_‐OCPCs, Fe^3+^_0.02_‐OCPCs, and Fe^3+^_0.04_‐OCPCs. All the nanoparticles represented hierarchical microarchitectures. However, the corona substructures varied along different *C*_Fe3_ *_+_*. Compared with Fe^3+^_0_‐OCPCs (Figure 2f), which exhibited a classic onion‐like structure with no evident porous corona because of the P2VP‐b‐PEO self‐assembly‐induced microphase separation,^[^ ^19a^ ^]^ Fe^3+^_0.02_‐OCPCs had a large number of approximately hexagonal pores (20–100 nm) closely packed at the corona (Figure 2g), the formation of which was caused by the Fe^3+^‐induced macrophase separation. As the *C_Fe_* ^3+^ increased to a larger extent (0.04 mm), the degree of macrophase separation was further enhanced, thus the pores in the corona became bigger, denser, and gradually lost their geometric regularity (Figure 2h). Taken together, the hierarchical microarchitecture of Fe^3+^‐OCPCs was precisely controlled by simply adjusting *C*_Fe_^3+^, including the number and size of the pores in the porous corona and the size and thickness of the multi‐layers in the onion‐like core substructure.

**Figure 3:**
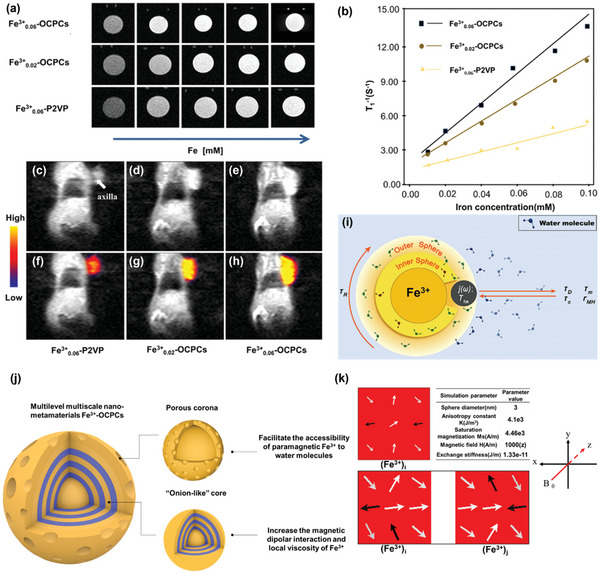
a) *T*_1_‐weighted MR images of aqueous solutions containing Fe^3+^_0.06_‐P2VP, Fe^3+^_0.02_‐OCPCs, and Fe^3+^_0.06_‐OCPCs. b) The longitudinal relaxivity (*r*_1_) of water protons in the presence of Fe^3+^_0.06_‐P2VP, Fe^3+^_0.02_‐OCPCs, and Fe^3+^_0.06_‐OCPCs. Plot of 1/*T*_1_ (*r*_1_) over [Fe^3+^] concentration of Fe^3+^_0.06_‐P2VP, Fe^3+^_0.02_‐OCPCs, and Fe^3+^_0.06_‐OCPCs. c–h) In vivo *T*_1_ contrast enhancement after 30 min intratumor injection of Fe^3+^_0.06_‐P2VP, Fe^3+^_0.02_‐OCPCs, and Fe^3+^_0.06_‐OCPCs in mice bearing subcutaneous axilla tumors. The dosage is 20 µmol kg^−1^ based on Fe^3+^. Arrow indicates the axilla. i) A depiction of time parameters and regions contributing to longitudinal relaxivity of Fe^3+^‐OCPCs. The time parameters are diffusional correlation time(*τ*_D_), rotational correlation time (*τ*_R_), the correlation time constant for the fluctuating magnetic dipole(*τ*_c_), the metal proton distance(*r*_MH_), and water residence time (*τ*_m_ for direct core interaction). *T*_1m_ refers to the *T*_1_ relaxation time of the inner sphere water molecules while *j*(*ω*) is a complex function of *r*_1_^OS^. The IS consists of water molecules interacting directly with Fe^3+^ in Fe^3+^‐OCPCs. The OS consists of water molecules transiently bound to the P2VP units which chelate Fe^3+^. The motion of water molecules through these two spheres determines *r*_1_ relaxivity. j) A schematic illustration of the microstructure‐biological performance relationship of Fe^3+^_0.06_‐P2VP nano‐metamaterials. k) Simulated magnetic spin states of 1 × 1 Fe^3+^ paramagnetic sphere ((Fe^3+^)_i_) arrangement and 1 × 2 Fe^3+^ paramagnetic spheres ((Fe^3+^)_i_ and (Fe^3+^)_j_) arrangement by using OOMMF program (Oxs uniform exchange‐field). The images were color‐mapped according to the angle of the spin deviation versus the external magnetic field (which is parallel to the *z*‐axis), white indicates nondeviated spins and black indicates highly canted spins.

The results clearly showed that, through the dual‐kinetic control strategy, the multilevel nano‐metamaterials Fe^3+^‐OCPCs were fabricated with controllable architectural and compositional complexity. Furthermore, from the compositional aspect, PEO and P2VP blocks are biocompatible and have been widely explored for biomedical applications such as drug delivery and cancer therapy.^[^ ^30^, ^31^ ^]^ Fe is an essential element for human beings and played a key role in metabolic processes. The safety evaluation experiment (Figure S7, Supporting Information) demonstrated that the viability of 1‐day‐old HeLa cells after 30 min treatment with Fe^3+^_0.06_‐P2VP, Fe^3+^_0.02_‐OCPCs, and Fe^3+^_0.06_‐OCPCs at different Fe^3+^ concentrations reached an average of 90%. Additionally, the Fe^3+^‐OCPCs have remarkable long‐term biostability and we found that the MR imaging effect exhibited no obvious decrease after 36 h co‐culturing as shown in Figure S8, Supporting Information. Therefore, compared to Gd^III^ complexes (Gd(DTPA)), clinical *T*_1_ MRI contrast agents that are challenged by nephrogenic systemic fibrosis and cranial nerve impairment, Fe^3+^‐OCPCs are expected to be more bio‐friendly. Fe^3+^ is an endogenous paramagnetic metal ion and possesses five unpaired S‐state electrons (indicating a large spin number), the spin relaxation of which closely matches with water proton spin relaxation, indicative of Fe^3+^‐OCPCs being used as an MRI contrast agent (**Figure** **3**).^[^ ^32^ ^]^ The magnetic property of Fe^3+^‐OCPCs was demonstrated by a vibrating sample magnetometer (VSM) and a superconducting quantum interference device (SQUID). In the revised manuscript, we corrected the explanation as “The field‐dependent magnetization (M‐H) curve of Fe^3+^‐OCPCs performed on VSM showed an ultralow magnetization with no coercivity and remanence (300 K).” When performed on ultra‐sensitive SQUID, a slight magnetic hysteresis was observed in the M‐H curve of Fe^3+^‐OCPCs (Figure S9, Supporting Information). Furthermore, unlike the magnetic iron oxide nanoparticles, which have the magnetic homogeneity disrupted and are widely used as *T*_2_‐weighted contrast agents, Fe^3+^ sustains the magnetic homogeneity and shied away from disturbing other anatomic backgrounds, thus providing efficient *T*_1_‐weighted contrast enhancement.^[^ ^33^, ^34^, ^35^ ^]^ Consequently, Fe^3+^‐OCPCs with high facet exposure and spatially controlled Fe^3+^ would provide high *T*_1_ contrast enhancement by accelerating the longitudinal relaxation of water molecules.

To test the speculation, we substituted P2VP‐b‐PEO block polymer with homopolymer P2VP and synthesized homogeneous Fe^3+^_0.06_‐P2VP nanoparticles as control (prepared from 0.06 mm Fe^3+^ and P2VP, about 520 nm, Figure S6, Supporting Information). Due to the strong chelation between Fe^3+^ and P2VP, Fe^3+^ is homogeneously distributed within Fe^3+^‐P2VP nanoparticles. We first used a 3.0 T MRI instrument to test the in vitro *T*_1_ contrast enhancement of Fe^3+^‐OCPCs and Fe^3+^_0.06_‐ P2VP. A series of aqueous solutions of Fe^3+^_0.02_‐OCPCs, Fe^3+^_0.06_‐OCPCs, and Fe^3+^_0.06_‐P2VP were prepared for characterization. We used the longitudinal relaxivity (*r*_1_) as a measure of contrast strength, which is defined as

(4) $$r_{1}=\left[\left(1/T_{1}\right)-\left(1/T_{1,0}\right)\right]/\left[{\mathrm{Fe}}^{3+}\right]$$

where [Fe^3+^] is the Fe^3+^ concentration, *T*_1_ represents the longitudinal relaxation times of the Fe^3+^‐OCPCs and Fe^3+^_0.06_‐P2VP solutions, and *T*_1,0_ represents the longitudinal relaxation time of water.^[^ ^36^ ^]^ Compared to homogeneous Fe^3+^_0.06_‐P2VP nanoparticles, the *T*_1_‐weighted MR images of Fe^3+^‐OCPCs were brighter (Figure 3a). In addition, the qualitative analysis (Figure 3b) showed that the molar relaxivity *r*_1_ per Fe^3+^ of Fe^3+^_0.02_‐OCPCs and Fe^3+^_0.06_‐OCPCs was 10.48 and 13.39 m m ^−1^ s^−1^, which were about 2.5‐ and 3.4‐folds higher than that of Fe^3+^_0.06_‐P2VP (4.21 m m ^−1^ s^−1^). The statistically significant increase in *r*_1_ indicated that *T*_1_ contrast enhancement was strengthened by the hierarchical architecture of Fe^3+^‐OCPCs, where *C*_Fe_^3+^ in Fe^3+^‐OCPCs provided a means to regulate.

To interpret the observed strong *r*_1_ boosting effect of Fe^3+^_0.06_‐OCPCs in comparison to the homogeneous Fe^3+^_0.06_‐P2VP, we employed the Solomon–Bloembergen–Morgan (SBM) theory, a quantum mechanical theory that is based on the theory of paramagnetic molecular model and could be used to discuss the paramagnetic metal chelation system at the molecular level.^[^ ^37^ ^]^ According to the SBM theory, the paramagnetic relaxation enhancement of Fe^3+^_0.06_‐OCPCs originated from both the inner‐sphere (IS) and the outer‐sphere (OS) mechanisms. The overall longitudinal relaxivity (*r*_1_) was the sum of the inner‐sphere relaxivity, *r*_1_^IS^, and the outer‐sphere relaxivity, *r*_1_^OS^ (Equation (5)).

(5) $$r_{1}=r_{1}^{\mathrm{IS}}+r_{1}^{\mathrm{OS}}$$

The *r*_1_^IS^ is derived from the water molecule spins present in the inner sphere of the Fe^3+^ ions, while the water molecules outside the inner sphere contributed toward the *r*_1_^OS^ (Figure 3j).^[^ ^33a^ ^]^ The *r*_1_^IS^ contribution to the longitudinal relaxivity *r*_1_ was calculated as

(6) $$r_{1}^{\mathrm{IS}}=\frac{P_{\mathrm{Fe}3+}}{c_{\mathrm{Fe}3+}}\frac{q}{T_{1m}+\tau_{m}}=1.8\times 10^{-5}\frac{q}{T_{1m}+\tau_{m}}$$

where *C*_Fe_^3+^ is the concentration of Fe^3+^ ions (mm); *P*_Fe_^3+^ is the mole fraction of Fe^3+^; *q* denotes the number of fast exchanging water molecules in the first coordination sphere of Fe^3+^ (inner sphere water molecules); *T*_1m_ and *τ*_m_ are the *T*_1_ relaxation time and residence lifetime of the inner sphere water molecules, respectively. The *r*_1_^OS^ contribution to the longitudinal relaxivity *r*_1_ is calculated as

(7) $$r_{1}^{\mathrm{OS}}=C\left[3j\left(\omega_{I}\right)+7j\left(\omega_{S}\right)\right]$$

where *C* is a constant and *j*(*ω*) is a complex function.

In this regard, the two substructures of Fe^3+^_0.06_‐OCPCs, including the onion‐like core and the porous corona, both contributed to the *r*_1_ relaxivity, either through *r*_1_^IS^ or *r*_1_^OS^, due to the spatial control of Fe^3+^ and high facet exposure. The onion‐like core accommodated multilayers of PEO, which intercalated between P2VP/Fe^3+^ layers, enabling the control of the spatial distribution of Fe^3+^. Water molecules, which tended to interact with Fe^3+^, became trapped in the interstices of the PEO layers. Thus, the onion‐like core increased the residence time *τ*_m_ by reducing the mobility of the outer‐sphere water molecules.^[^ ^33b^ ^]^ The relationship between *τ*_m_ and *j*(*ω*) is given as

(8) $$\begin{array}{ccc} j\left(\omega \right) & = & \left[4+{\left(i\omega \tau_{D}+\frac{\tau_{m}}{T_{1e}}\right)}^{1/2}\right]/\left[4+4{\left(i\omega \tau_{D}+\frac{\tau_{D}}{T_{1e}}\right)}^{1/2}\right.\\ & & \left.+\frac{16}{9}\left(i\omega \tau_{D}+\frac{\tau_{D}}{T_{1e}}\right)+\frac{4}{9}{\left(i\omega \tau_{D}+\frac{\tau_{D}}{T_{1e}}\right)}^{3/2}\right] \end{array}$$

where *T*_1e_ is the electronic longitudinal time of Fe^3+^. As the correlation time *τ*_D_ increased, the *j*(*ω*) increased as well (Equation (8)), which further boosted the value of *r*_1_^OS^ (Equation (7)). Besides, the confinement of Fe^3+^ in P2VP/Fe^3+^ layers also reduced the ability of Fe^3+^ to rotate freely, and thus, increased the characteristic tumbling time *τ*_R_, which eventually led to an increase in the correlation time of *τ*_c_.

(9) $$\frac{1}{\tau_{c}}=\frac{1}{T_{1e}}+\frac{1}{\tau_{m}}+\frac{1}{\tau_{R}}$$

where *τ*_c_ is the correlation time, as the time constant for the fluctuating magnetic dipole that mediates proton relaxation, *τ*_m_ is the residence time of the water molecules in the complex; and *τ*_R_ is the tumbling time. The increase of *τ*_c_ would further reduce *T*_1m_ (*T*_1_ relaxation time), which could be described as^[^ ^33b^ ^]^

(10) $$\frac{1}{T_{1m}}=\frac{2}{15}\left(\frac{\mu_{0}}{4\pi }\right)\frac{\gamma_{H}^{2}g_{e}^{2}\mu_{B}^{2}S\left(S+1\right)}{r_{\mathrm{MH}}^{6}}\left[\frac{3\tau_{c}}{1+\omega_{H}^{2}\tau_{c}^{2}}\right]$$

where *γ*_H_ is the gyromagnetic ratio of a proton (*γ*_H_ = 2.675 × 10 ^+^ ^8^ T^−1^ s^−1^); *g^e^*, the electron g‐factor (*g* = 2); *S*, the total electron spin of the Fe^3 +^ ion (*S* = 5/2); *µ*_B_, the Bohr magneton (*µ*_B_ = 9.274 × 10^−24^ JT^−1^); *µ*_0_, the permeability of vacuum (*µ*_0_ = 1.257 × 10^−6^ NA^−2^); *r*_MH_, the metal proton distance; and *ω*_H_, the proton Lamor frequency (*ω*_H_ = 658(2*πv*)).

According to Equation (6), reducing *T*_1m_ would result in a high value of *r*_1_^IS^. Therefore, the onion‐like substructure boosted both *r*_1_^IS^ and *r*_1_^OS^ and ultimately *r*_1_, due to the spatial control of Fe^3+^.

Besides the onion‐like core, the porous corona contributed positively to *r*_1_^IS^ as well. The high facet exposure that resulted from the porous corona of Fe^3+^_0.06_‐OCPCs facilitated the accessibility of paramagnetic Fe^3+^ ions to water molecules (Figure 3j), thus reducing Fe^3+^‐proton distance *r*_MH_ and increased *r*_1_^IS^ (Equation (10)).^[^ ^33b^ ^]^ Taken together, the hierarchical architecture of Fe^3+^_0.06_‐OCPCs, including the onion‐like core and the porous corona, enhanced *T*_1_ contrast compared to that of homogeneous Fe^3+^_0.06_‐P2VP in the control group.

*C*_Fe_^3+^ in Fe^3+^‐OCPCs also provided a means to regulate the *T*_1_ contrast enhancement by adjusting the inner‐sphere contribution. Compared with Fe^3+^_0.02_‐OCPCs, Fe^3+^_0.06_‐OCPCs had large pore sizes and pore numbers in their corona, which meant a high facet exposure. Therefore, the average value of *r*_MH_ (the metal proton distance) in the Fe^3+^_0.06_‐OCPCs group was smaller than that in the Fe^3+^_0.02_‐OCPCs group, leading to a higher *r*_1_^IS^ according to Equations (10) and (6). As a result, the *r*_1_ value of Fe^3+^_0.06_‐OCPCs was larger than that of Fe^3+^_0.02_‐OCPCs (Equation (5)), which was in consistence with our experimental data (Figure 3a). Therefore, the architectural complexity of Fe^3+^‐OCPCs allowed for the controllable *T*_1_ contrast enhancement.

Additionally, the micromagnetic simulation and rotational dynamics analysis of paramagnetic Fe^3+^ ions were conducted to further manifest the microstructure‐biological performance relationship of Fe^3+^‐OCPCs nano‐metamaterials. We observed that compared to homogeneous Fe^3+^‐P2VP nanoparticles, the confinement of Fe^3+^ ions in the hierarchical microarchitecture of Fe^3+^‐OCPCs changed two important factors, the dipolar interaction rate between Fe^3+^ ions (*R*_Fe3+, dip_) and rotational Brownian motion rates of Fe^3+^ ions (*R*_Fe3+, rot_), thus enhancing *r*_1_ relaxivity (Figure 3j,k and Figures S10–S12, Supporting Information).

Having confirmed that Fe^3+^‐OCPCs exhibited great *T*_1_ contrast enhancement in vitro, we next investigated whether Fe^3+^‐OCPCs had high *T*_1_ contrast enhancement in vivo. A 3.0 T MRI instrument was then applied to conduct in vivo MR imaging. The tumor model was established by subcutaneously injecting HeLa cells (1.3 × 10^6^) into the subaxillary of the female Balb/C mice (6 weeks old). When tumor volume reached about 5 mm^3^, three groups of randomly selected mice were intratumorally injected with Fe^3+^_0.02_‐OCPCs, Fe^3+^_0.06_‐OCPCs, and Fe^3+^_0.06_‐P2VP. Figure 3c–h shows the in vivo *T*_1_ contrast enhancement of Fe^3+^‐OCPCs and Fe^3+^_0.06_‐P2VP. Compared to homogeneous Fe^3+^_0.06_‐P2VP, Fe^3+^‐OCPCs groups exhibited significantly higher *T*_1_ contrast enhancement, in accordance with the in vitro results (Figure 3i). Furthermore, we investigated high‐field MR imaging of Fe^3+^‐OCPCs using clinical contrast agents (Gd(DTPA)) as a control. More interestingly, compared with a low magnetic field (3 T) MRI system (Figure 3c–e), high‐field (7 T) strength improved the signal‐to‐noise ratio of Fe^3+^‐OCPCs and thus allowed the acquisition of MR images with higher resolution. As shown in Figure S13, Supporting Information, we further investigated the MR imaging performances of Fe^3+^‐OCPCs in zebrafish embryos (98 h after fertilization). At 7 T MR scanning, compared to Gd(DTPA) group, more detailed information about the microscopic structures was visualized in the transverse planes in groups i.v. injected with Fe^3+^‐OCPCs (98 h after fertilization). The study of the mechanism of Fe^3+^‐OCPCs enhanced high‐field (7 T) MRI is still ongoing.

## 3 Conclusions

In summary, this work presented a novel nano‐metamaterial Fe^3+^‐OCPCs, which not only possess multilevel microarchitectures with both characteristic size and whole size at the nanoscale but also exhibit a superior *T*_1_ contrast effect in MR imaging. In our work, two separate kinetic pathways, BCP self‐assembly and droplet self‐emulsifying, are essential to the manufacturing of hierarchical nano‐metamaterials. Due to the two mutually independent kinetic processes, the unusual multilevel microarchitecture, including a porous corona at the architectural scale and a multilayered onion‐like core at the molecular scale, was observed in Fe^3+^‐OCPCs. This hierarchical structure altered the *T*_1_ parameters, including, *τ*_m_, *τ*_R_, and *τ_D_*, and potentially others, by reducing the ability of the Fe^3+^ to tumble and by decreasing the mobility of the water molecules, thus generating a coordinative effect to improve significantly *T*_1_ relaxivity. These results demonstrated that in addition to sizes, shapes, and material properties, the hierarchy was another important factor that needs considering in using nanoparticles for biomedical imaging. Thus, we first synthesized nano‐metamaterials using a dual‐kinetic controlled strategy and manifested a microstructure‐biological performance relationship of nano‐metamaterials. It will be helpful to further develop nano‐metamaterials based on the established dual‐kinetic strategy, with their microarchitectures and applications regulated through adjusting different reaction constituents including BCP, solvent, ions, and nanoparticles. Furthermore, multiple agents, such as functional nanoparticles, active molecules, and drugs, might also be introduced into the hierarchical microstructures of nano‐metamaterials to generate multifunctional systems that have both imaging and therapeutic capabilities.^[^ ^38^ ^]^ However, the kinetic instability limits our approach to conducting large‐scale production of nano‐metamaterials. Our future research will focus on improving the controllability of our approach to obtain nano‐metamaterials with smaller sizes to facilitate the detection, clearance, and imaging of physiologically malfunctional or pathological tissues. Nevertheless, we believe that more productive synthesis protocols for nano‐metamaterials with different hierarchical microarchitectures will be inspired by our approach. Meanwhile, by using well‐defined Fe^3+^‐OCPCs as the modeling material, a microstructure‐biological performance relationship was manifested in nano‐metamaterials and new insights into the understanding of the structure‐function relationship of nano‐metamaterials will also be inspired. New classes of diagnostic‐therapeutic agents that act by virtue of their microscale hierarchy will soon emerge.

## Conflict of Interest

The authors declare no conflict of interest.

## Supporting information

Supporting Information

## Data Availability

The data that support the findings of this study are available in the supplementary material of this article.
