# Supplementary material for: A Dual‐Kinetic Control Strategy for Designing Nano‐Metamaterials: Novel Class of Metamaterials with Both Characteristic and Whole Sizes of Nanoscale
Source: Adv Sci (Weinh). 2022 Nov 15;10(4):2205595. doi: 10.1002/advs.202205595 (PMC9896071; doi:10.1002/advs.202205595)
Supplement: Supplementary file 1 — Supporting Information [file ADVS-10-2205595-s001.pdf]

ADVANCED SCIENCE
Open Access

Supporting Information

for *Adv. Sci.*, DOI 10.1002/advs.202205595

A Dual-Kinetic Control Strategy for Designing Nano-Metamaterials: Novel Class of Metamaterials with Both Characteristic and Whole Sizes of Nanoscale

Guanhua Xu, Mengmeng Li, Qiyue Wang, Feng Feng, Qi Lou, Yi Hou, Junfeng Hui, Peisen Zhang, Li Wang, Li Yao, Shijie Qin, Xiaoping Ouyang, Dazhuan Wu, Daishun Ling* and Xiuyu Wang**

Supplementary Materials for A dual-kinetic control strategy for designing Nano-metamaterials ：novel class of metamaterials with both characteristic and whole sizes of nanoscale

Guanhua Xu, Mengmeng Li, Qiyue Wang, Feng Feng, Qi Lou, Yi Hou, Junfeng Hui, Peisen Zhang, Li Wang, Li Yao*, Shijie Qin, Xiaoping Ouyang, Dazhuan Wu, Daisun Ling* and Xiuyu Wang*

Experimental Section

Chemicals and reagents

The block copolymer (BCP) of PEO-*b*-P2VP was derived from Polymer Source, Inc. Canada. Detailed information of PEO-*b*-P2VP was listed in Table S1. Ferric chloride hexahydrate ($FeCl_3{\cdot}6H_2O$), hydroxylamine hydrochloride, and phenanthroline was purchased from Beijing Innochem Co., China. Aqueous surfactant Poly(vinyl alcohol) (PVA, Mw =13-23k, 87-89% hydrolyzed) was purchased from Sigma-Aldrich. Phosphate-buttered saline (PBS, 20 mM potassium phosphate pH 7.4, 150 mM NaCl) was obtained from HyClone/Thermofisher (Beijing, China). Dimethyl formamide (DMF, ACS certified) and sodium hydroxide (NaOH, ACS certified) with analytic grade was purchased from Beijing Reagents Co., China. Fetal Bovine Serum (FBS) was purchased from Gibco (Thermo Fisher Scientific inc.), Penicillin and streptomycin, 1640 medium and Trypsin were obtained from HyClone/Thermofisher (Beijing, China). HeLa cells were obtained from Yan group (Life Sciences Institute, Zhejiang University, Hangzhou). Deionized water was used in all experiments. All of the materials were used without further purification.

Fe_x-OCPCs Generation

To generate monodisperse emulsion droplets of well-controlled sizes, we constructed microfluidic devices by assembling glass capillaries on a glass slide. The inner capillary was tapered by a Sutter micropipette pullers P-1000 (Sutter Instrument Co., U.S.A.) and was calibrated under an optical microscope to reach the desired orifice diameter of about 5-30 μm. Then the tapered end of the inner capillary was coaxially nested into the outer square capillary with an inner diameter of 1.05 mm (Vitro Com, Inc.), and sequentially assembled on a glass slide. The dispersed phases flowed through the inner capillary; and the continuous phase flowed via the interstices between the inner and the outer capillaries. Typically, a mixture

solvent of DMF and CH_2Cl_2 (volume ratio of 1:10) containing PEO-*b*-P2VP (0.01 mg) and $FeCl_3 \cdot 6H_2O$ with a varying molar ratio of vinyl pyridine/Fe^{3+} of 60, 40, and 20 were prepared by stirring for at least 2 h to give a homogeneous solution. Subsequently, 4 μM of NaOH was added to the copolymer solutions, and then the mixture was stirred overnight to reach equilibrium. The prepared mixture solution was used as dispersed phase. The continuous phase was an aqueous solution of PVA with a concentration of 0.4 wt%. The two phases were pumped to the microfluidic device by a syringe pump (Harvard PHD 2000 Series); and in the sequential coflow regime, as shown in **Figure 1**a in the main text, the dispersed phase broke into highly monodisperse emulsion droplets because of interfacial tension. The droplet diameters could be readily tuned from 15 to 80 μm by the size of the orifice and the flow rates of the two phases. Immediately after formation, the micron-sized monodispersed droplets were sequentially extruded through a filter membrane with a size of 0.45 μm. The nano-sized droplets prepared through the second filter stage were too small to be studied in suit during the solidification process. Instead, we monitored the structural evolution of micron-sized droplets as indirect evidence to study the structural and morphological evolution of the nano-sized droplets during the solvent evaporation. Although they were different in the dimensional sizes, both droplets were in non-equilibrium, and could be metastable with the droplets retaining their stability for extended periods of experimental time, because their interfaces were stabilized by a PVA surfactant.[1] The micron-sized droplets through first microfluidic stage were collected in a homemade container at room temperature. The structural and morphological evolution of the prepared droplets were carefully observed during the solvent evaporation. Depending on the geometry and the quantity of droplets, the evaporation of solvent took tens of minutes to hours, and we observed that the nano-sized droplets solidified faster just within 1 minute. Finally, after the impurities were removed by dialysis purification, Fe^{3+}-OCPCs nanoparticle solution was obtained and placed at 4 ℃ for standby.

Characterization

Real time structural evolution of the emulsion droplets containing Fe^{3+}/PEO-*b*-P2VP was monitored using Olympus IX71 inverted optical microscope in the bright-filed mode. The resulting hierarchical Fe^{3+}-OCPCs structures were observed by transmission electron microscopy (TEM) images (HD-2000, Hitachi Ltd., Japan), operating at an acceleration voltage of 60 kV. Typically, Fe^{3+}-OCPCs were welded into a frozen paraffin before cut into slices with a certain thickness using a Bühler high-speed diamond-blade saw equipped with water cooling, carefully transferred onto a copper TEM grid with a carbon supporting film

and allowed to dry in air and at room temperature for 1 day. The P2VP domains which were chelated by Fe^{3+} appeared dark in the TEM observations due to the high atomic number of Fe. I_2 vapor was employed to observe the phase separation structures in Fe^{3+}_0-OCPCs block copolymer nanoparticles because they could selectively react with the P2VP domains in Fe^{3+}_0-OCPCs. The suspensions of Fe^{3+}_0-OCPCs particles (0.5 mL) were stained with I_2 vapor for 2 h at room temperature. After staining, the stained particles were centrifuged (12000 rpm., 5 ℃, 15 min) and washed with deionized water to eliminate excess I_2. After washing, the stained particles were redispersed in pure water with ultrasonication. Suspensions of the stained particles in water were dropped onto a carbon membrane surface placed on a Cu mesh and dried at room temperature. The phase separation structures in the particles were then observed by scanning transmission electron microscopy (STEM; HD-2000, Hitachi Ltd., Japan).The morphologies of Fe^{3+}-OCPCs were observed by scanning electron microscopy (SEM) which was carried out on a FEI NOVA Nano400 instrument at an accelerating voltage of 60 kV. To prepare sample for SEM, the Fe^{3+}-OCPCs on the glass substrates were coated with a thin layer of gold. Dynamic light-scattering measurements were carried out using Zetasizer, Malvern company, UK. equipped with a He-Ne Laser (632.8nm) for measuring the hydrodynamic size of the resultant Fe^{3+}-OCPCs. Small-angle X-ray scattering (SAXS) measurements were performed on a Nanostar U small-angle X-ray scattering system using CuKa radiation ($\lambda_{CuK\alpha}$= 1.5418 Å). The SAXS data at room temperature were collected over a 2θscattering range 0.5 -2.8° with a step 0.02. The magnetic properties were measured using vibrating sample magnetometer (VSM) and superconducting quantum interference device (SQUID). VSM data were collected on the BKY-400 magnetometer with a sensitivity of 2×10^{-5} emu. SQUID magnetometry was performed using a Quantum Design MPMS with dc detection from Oxford Instruments. The pore size distribution profile of Fe^{3+}-OCPCs was measured by nitrogen sorption at 77.3 K using a Micromeritics ASAP 24204 volumetric adsorption analyzer. Fe^{3+}-OCPCs were degassed offline at 393 K for 12 h under dynamic vacuum (10-5 bar) before analysis, followed by degassing on the analysis port under vacuum, also at 393 K. An inductively coupled plasma mass spectrometry (ICP-MS) Thermo iCAP RQ was used to measure the Fe and N content of the samples. The samples were digested by using HCl/HNO_3 (3/1 v/v). Fourier transform infrared (FTIR) spectra were obtained at room temperature using a Bruker EQUINOX 55 FTIR spectrometer.

Determined Fe^{3+} concentration

Whether the biological experiments or subsequent T_1-weighted MR image tests, we needed to determine accurately the Fe^{3+} concentration in Fe^{3+}-OCPCs and Fe^{3+}-P2VP nanoparticles. The

Fe^{3+} concentration of the investigated solutions of the Fe^{3+}-OCPCs and Fe^{3+}-P2VP was determined by the procedure phenanthroline spectrophotometry. There were two principles of this method: first, according to Lambert Beer law: $A = \varepsilon\ bC$, A was the absorbance of a colored substance, and C was the concentration of the substance. When the incident light ε and the optical path b was constant, we could see that A was directly proportional to C. Second, phenanthroline (phen) could form a stable orange red complex with iron ions within a certain pH range. The reaction equation was as follows: $Fe^{2+} + 3(phen) = Fe(phen)_3{}^{2+}$. Therefore, this method needed to draw a standard curve first, then tested Fe^{2+} according to the standard curve.

We first prepared iron standard solution. A small amount of deionized water was added to 0.484 mg $FeCl_3 \cdot 6H_2O$ to dissolve it completely. After full dissolution, drained it with a glass rod and transferred the mixture to a 1000 mL volumetric flask, and finally diluted the mixture to the scale with an appropriate volume of deionized water. Then, we prepared 0.15% phenanthroline aqueous solution. 5-10 mL of 95% ethanol solution was added to 1.5 g phenanthroline. After full dissolution, drained the mixture with a glass rod and transferred it to a 1000 ml volumetric flask, and finally diluted the mixture to the scale with an appropriate volume of deionized water.

With all the prework completed, 1 mL hydroxylamine hydrochloride solution was added into iron standard solution (0 ml, 2 ml, 4 ml, 6 ml, 8 ml, 10 ml, 12 ml and 14 ml of 10 ml).Then, 2 mL 0.15% phenanthroline solution was added into the mixture, which appeared a colorless and transparent solution. After 10 minutes, 5 mL HAC NaAc buffer solution (pH = 4.6) was added into the prepared solution, shook well and stood for 10min. At this time, the liquid appeared transparent orange. Finally, drained the colorful mixture with a glass rod, and diluted to the 50 mL with deionized water. During the UV-Vis spectrum test, set the wavelength range between 350-700nm, and measured the UV-Vis spectrum of each solution. Taking the absorption value of UV-Vis spectrum at 510 nm, the standard curve of iron concentration and correlation coefficient were obtained.

Fe^{3+}-OCPCs or Fe^{3+}-P2VP containing solutions were mixed in a 1:4 ratio with 69% HNO_3 and 31% HCl and heated in sealed vials at 120 °C overnight to yield a solution of Fe^{3+} aqua ion. 2 mL 0.15% phenanthroline solution was added into Fe^{3+} solution. After 10 minutes, 5mL HAC NaAc buffer solution (pH=4.6) was added into the prepared mixture, shook well, and stood for 10 min. At this time, the liquid appeared transparent orange. Finally, drained the colorful mixture with a glass rod, and diluted to the 50 mL with deionized water for further UV-Vis tests. Measured the UV-Vis spectrum of each solution at absorption value at 510nm,

and calculated the content of Fe^{3+} according to the standard Fe^{3+} curve.

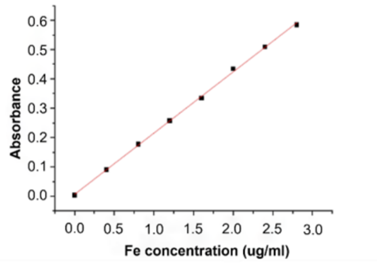

The relation curve of Ultraviolet (UV) absorbance and concentration of Fe^{3+}. Y=0.20884X+0.0065，correlation coefficient is 0.999

Light microscopy

To examine the structural and morphological evolution of the prepared droplets during the solidification process, phase contrast images of static bright field droplets were captured at different points of time, using the Olympus IX71 inverted optical microscope equipped with 60x phase-contrast objective. All image data shown were representative samples from three random fields.

Cell culture

HeLa cells were obtained from Yan group (Life Sciences Institute, Zhejiang University, Hangzhou). HeLa cells were maintained in DMEM supplemented with 10% FBS, penicillin (100 U/ml) and streptomycin (100 mg/ml) at 37 ℃ with 5% CO_2. HeLa Cells were passaged every 48 h and split 1/2. For experiments, cells were incubated with fresh medium for 24 h to allow cell adhesion. Then the culture medium was replaced by fresh medium containing the nanoparticles, after which the experiments were carried out. The prepared anoparticles were sterilized prior to the addition to the culture medium by filtration with a sterile membrane filter of 0.65 μm pore diameter.

Cytotoxicity.

For the determination of cellular viability, HeLa cells seeded in 96-well plates at 5×10^4 cells per well and and cultured in 1640 culture medium overnight. Then, the culture medium was replaced by fresh medium containing a series of concentrations of $Fe^{3+}_{0.06}$-OCPCs (the Fe^{3+} concentration is 0, 2, 10, 20 μM), $Fe^{3+}_{0.06}$-P2VP and $Fe^{3+}_{0.02}$-OCPCs as the control group.

After another 30 min of treatment, the supernatant containing the excrescent nanoparticles was decanted, and the residual nanoparticles were washed by the fresh media and the proportion of living cells was evaluated by CCK-8 assay. 10 μL CCK-8 was added to each well, followed by 2.5 h incubation at 37 °C. After this period, the OD value of each well was read out using a Microplate Reader (Microplate Titre Infinite F200, TECAN Spectra) at 450 nm. At each concentration, three individual experiments were carried out.

Biostability in Cell Culture Medium

L929 cell line was seeded in 96-well plates at a density of 10^4 cells per well with 100 μL of culture medium (DMEM supplemented with 10% FBS, penicillin (100 IU/mL) and streptomycin (100 mg/mL)). The cells were grown in humidified tissue culture incubator (SANYO) at 37 °C with 5% CO_2 for 24 h. Then, the culture medium was collected and filtrated using a 0.20 μm Millex1-GS filter (Merck Millipore Ltd., Tullagreen, Ireland). Fe^{3+}-OCPCs were mixed with cell culture medium in a cone-shaped tube. After t=0 (a), 6 (b), 12 (c), 24 (d), 36 (e), 48 (f) h immersion of the nano-metamaterials, the *in vitro* T_1 contrast enhancement of the filtrate was tested by a 3.0 T MRI instrument (Bruker)

***T_1* Relaxometric Measurements**

The relaxivity measurements were carried out on a 3 T clinical MRI instrument (Philips Achieva 3.0 T TX). Observed longitudinal relaxation rates ($R_{1obs} = 1/T_{1obs}$) values were determined by inversion recovery at 21.5 MHz and 25 °C. Temperature was controlled with a Stelar VTC-91 airflow heater and the temperature inside the probe checked with a calibrated RS PRO RS55-11 digital thermometer. Data were acquired using a recovery time of ≥5 × T_1 and with 2 scans per data point. The absolute error in R_{1obs} measurements was less than 1%. A series of aqueous solution of Fe^{3+}-OCPCs or Fe^{3+}-P2VP in 2 mL Eppendorf tubes were prepared. The iron concentration of the investigated solutions of the Fe^{3+}-OCPCs and Fe^{3+}-P2VP was determined by the above descripted method. Then, the R_{1obs} of the various solution was measured at 298 K and 21.5 MHz could be determined using the equation $R_{1obs} = R_{1d} + r_{1p}^{Fe}$ [Fe], where R_{1d} is the diamagnetic contribution (0.48 s^{-1}) and r_{1p}^{Fe} [Fe] is the Fe^{3+} aqua ion relaxivity (18.47 mM^{-1} s^{-1}) under the same experimental conditions. The parameters were ste as echo time (TE) = 5.13 ms, repetition time (TR) = 300 ms, and number of excitations (NEX) = 8. Longitudinal relaxivity (r_1) values reported in the main context in Figure 3b were calculated as slope of the lines correlating observed relaxation rates measured at pH = 7.4, 298 K (1 T) as a function of Fe^{3+} concentration.

T_1*-weighted MR Imaging of Tumor *in Vivo

The xenografted tumor model used was established upon subcutaneous injection of HeLa cells (~5 ×10^6) into axilla of female BALB/c nude mice (4~6 weeks old). When the tumors had developed to about 1-4 mm in diameter, the mice were randomly divided into four groups (n = 6 mice per group) in a way to minimize weight and tumor size differences among the groups. Then, PBS, $Fe^{3+}_{0.02}$-OCPCs, $Fe^{3+}_{0.06}$-OCPCs (the Fe^{3+} concentration is 0, 2, 10, 20 μM), or Fe^{3+}-P2VP (20 μM, 200 μL) was intratumorally administered. After 40 minutes, xenografted tumor mice were anesthetized and MR images were acquired on with a Bruker BioSpec 3T MRI GmbH scanner (Bruker Biospin, Ettlingen, Germany), equipped with a 30 mm 1H quadrature coil at room temperature (RT = 21 °C). T_1-weighted images were acquired at designed time points. T_1-weighted images were acquired using a standard multislice multiecho sequence with the following parameters: repetition time (TR) 300 ms; echo time (TE) 11 ms; flip angle = 180°, number of averages = 4, field of view (FOV) 100 mm × 100 mm, slice thickness 1 mm; matrix size, 256 × 256. A series of T_1-weighted MSME scans were acquired before and after the intratumoral administration of the contrast agents after 40 minutes. The T_1 contrast enhancement (T_1 enh %) was calculated as follows:

$$\mathrm{SE}\ \% = \frac{\left(\frac{\mathrm{SI(ROI)post}}{\mathrm{SI(tumor)post}}\right) - \left(\frac{\mathrm{SI(ROI)pre}}{\mathrm{SI(tumor)pre}}\right)}{\left(\frac{\mathrm{SI(ROI)pre}}{\mathrm{SI(tumor)pre}}\right)} \times 100$$

where SI(ROI)post and SI(ROI)pre were the signal intensities in the regions of interest (both normalized by dividing for signal intensity in tumor taken as reference) post- and pre-injection of contrast agents.

High-field MRI examination

Each contrast agent (Gd(DTPA), $Fe^{3+}_{0.06}$-P2VP or $Fe^{3+}_{0.06}$-OCPCs) was adjusted to a final concentration of 0.1 M in 1× Danieau solution [5mM Hepes, pH 7.6, 58 mM NaCl, 0.7 mM KCl, 0.4 mM $MgSO_4$, and 0.6 mM $Ca(NO_3)_2$] and injected into the zebrafish embryo from one-cell to four-cell stages using the Eppendorf FemtoJet microinjector system (about 1 μL solution is injected per embryo). After injection, normal development of the embryos at 98 h was observed under a Nikon SMZ-U stereomicroscope. To image zebrafish embryos, a specific sample holder was designed: PDMS substrates were prepared using a SYLGARD184 Silicone Elastomer Kit. Briefly, an elastomer base and a curing agent were thoroughly mixed in a ratio (w/w) of 10 : 1 and degassed under vacuum for 30 min. Then, the mixture was

poured into a designed mould with a square well (well size 4 mm×4 mm) in the center, and then cured at 60 °C for 24 h. After that, the square well was hydroxylated using a plasma cleaner for 25 s. Then melted aqueous agarose solution was poured in the square well and a zebrafish embryo was placed in the submerged agarose well. When the agarose gel was completely formed, MR images were obtained on a 7 T Biospec system (Bruker Biospin, Ettlingen, Germany) using a birdcage coil of 72 mm inner diameter for radio frequency transmitting and a surface coil of 15mm diameter for receiving (Bruker Biospin). The T_1-weighted images were acquired with TR/TE 100/14 ms and 6 NEX.

Additional Characterization Data

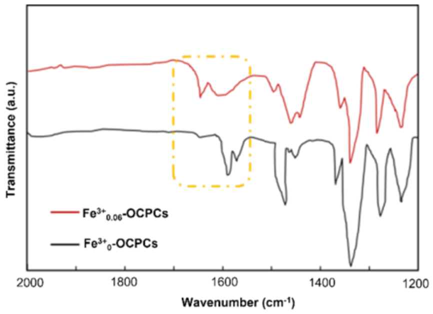

Figure S1. FT-IR spectra of the Fe^{3+}_{0}-OCPCs (black line) and $Fe^{3+}_{0.06}$-OCPCs (red line).

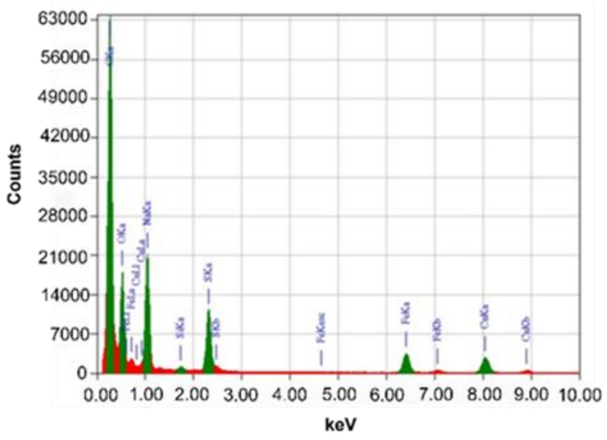

Figure S2. The ICP spectrum of $Fe^{3+}_{0.06}$-OCPCs.

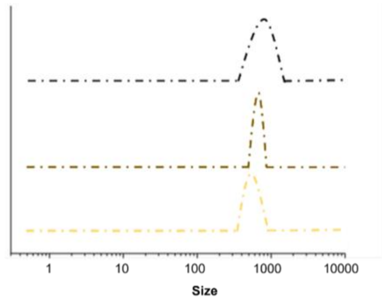

Figure S3. DLS data for Fe^{3+}-OCPCs with different Fe^{3+} concentration. The diameters were measured to be 500 nm, 540 nm and 560 nm for concentration 0.02 mM (yellow), 0.04 mM (brown) and 0.06 mM (black) samples, respectively.

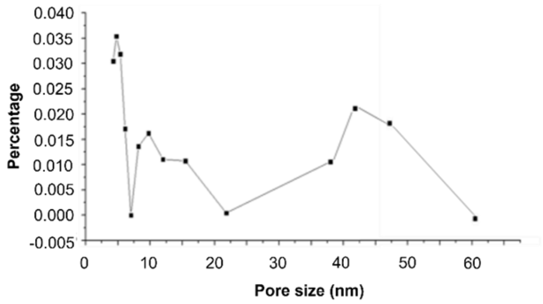

Figure S4. The pore size distribution profiles of $Fe^{3+}_{0.06}$-OCPCs.

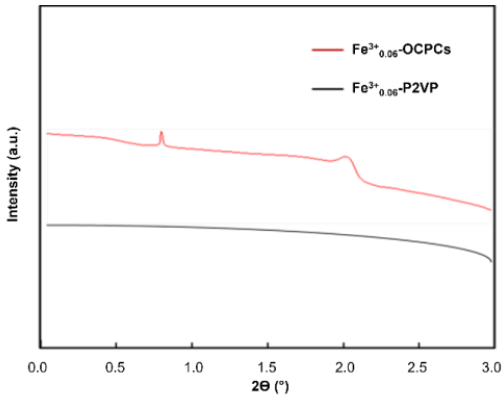

Figure S5. Small angle X-ray scattering (SAXS) of the $Fe^{3+}_{0.06}$-P2VP (black line) and $Fe^{3+}_{0.06}$-OCPCs (red line).

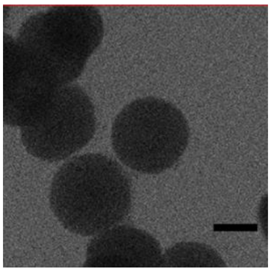

Figure S6. TEM image of homogeneous Fe^{3+}-P2VP nanoparticles. The bar is 400 nm.

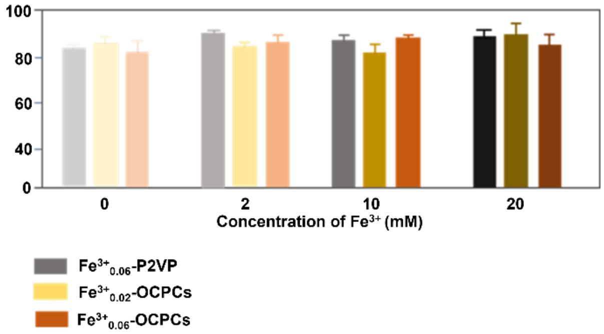

Figure S7. Viability of 1 day-old HeLa cells after 30 minutes treatment with $Fe^{3+}_{0.06}$-P2VP, $Fe^{3+}_{0.02}$-OCPCs and $Fe^{3+}_{0.06}$-OCPCs at different Fe^{3+} concentrations. The data are average of triplicates, and the error bars indicate the standard deviations.

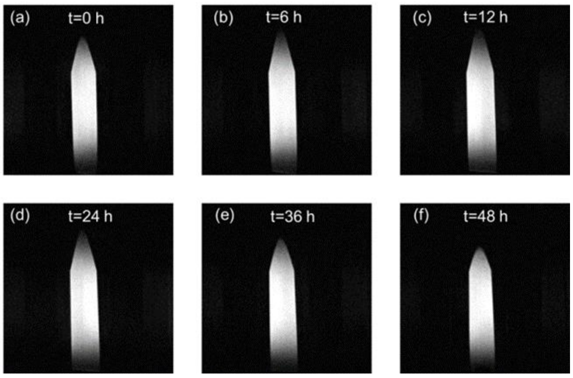

Figure S8. T_1-weighted MR images of Fe^{3+}-OCPCs immersed in cell culture medium after 0 (a), 6 (b), 12 (c), 24 (d), 36 (e), 48 (f) h.

We firstly evaluated the biostability of Fe^{3+}-OCPCs to ensure the *in vivo* applications. The filtrate of fibroblast cell culture medium was used to mimic cell microenvironment in normal tissues. We mixed Fe^{3+}-OCPCs with the filtrate of cell culture medium in a cone-shaped tube. After t=0 (a), 6 (b), 12 (c), 24 (d), 36 (e), 48 (f) h immersion of the nano-metamaterials, the T_1-weighted MR imaging of the filtrate was tested. As shown in **Figure S8**, we found that the MR imaging exhibited no obvious decrease after 36 h co-culturing, indicating that our Fe^{3+}-OCPCs were biostable in culture medium.

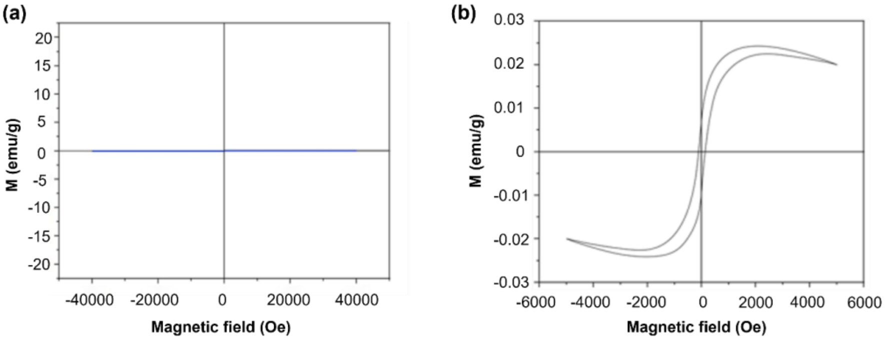

Figure S9. Magnetic hysteresis loops of Fe^{3+}-OCPCs characterized by VSM (a) and SQUID (b). The field-dependent magnetisation (M-H) curve of Fe^{3+}-OCPCs performed on VSM showed an ultralow magnetisation with no coercivity and remanence (300 K). When performed on ultra-sensitive SQUID, a slight magnetic hysteresis was observed in the M-H curve of Fe^{3+}-OCPCs.

Discussion for the enhanced longitudinal relaxivity of Fe^{3+}-OCPCs **based on SBM theory**

The mole fraction P_{Fe} in equation 6 is given as

$$P_{\mathrm{Fe}} = \frac{m_{\mathrm{Fe}}}{m_{\mathrm{Fe}}+m_{\mathrm{H_2O}}} \approx \frac{m_{\mathrm{Fe}}}{m_{\mathrm{H_2O}}} \quad \text{(SI-1)}$$

where m_{Fe} and m_{H2O} are respectively the number of moles for Fe^{3+} and water in one liter of solution.[2] For the water molecules m_{H2O}=55.56 (55.56 M). For Fe^{3+} with a concentration [Fe]= 0.06 mM, it follows m_{Fe}= 60×10^{-6} M. Thereby for the present case

$$P_{\mathrm{Fe}} \approx \frac{60\times10^{-6}}{55.56} = 1.08\times10^{-6} \quad \text{(SI-2)}$$

and the ratio P_{Fe}/[Fe] would be

$$P_{\mathrm{Fe}}/[\mathrm{Fe}] \approx \frac{1.08\times10^{-6}}{60\times10^{-3}} = 1.8\times10^{-5} \quad \text{(SI-3)}$$

being [Fe] expressed in mM, for a r_1 measured in $\mathrm{mM^{-1}s^{-1}}$

Whilst τ_{m} is an intrinsic property of the complex, the characteristic time $T_{1\mathrm{m}}$ can be expressed through the Solomon-Bloembergen relation as

$$\frac{1}{T_{\mathrm{m}}} = \frac{2}{15}\frac{C_{DD}}{r_{\mathrm{FeH}}^6}\left[\frac{7\tau_{c2}}{1+\omega_{\mathrm{S}}^2\tau_{\mathrm{c2}}^2}+\frac{3\tau_{c1}}{1+\omega_{\mathrm{I}}^2\tau_{\mathrm{c1}}^2}\right]+\frac{2}{3}S(S+1)\left(\frac{A}{\hbar}\right)^2\left[\frac{\tau_e}{1+\omega_{\mathrm{S}}^2\tau_{\mathrm{c2}}^2}\right] \quad \text{(SI-4)}$$

with the constant C_{DD} given by

$$C_{\mathrm{DD}} = \gamma_{\mathrm{I}}^2 g^2 \mu_{\mathrm{B}}^2\left(\frac{\mu_0}{4\pi}\right)^2 S(S+1) \quad \text{(SI-5)}$$

where γ_{I} is the gyromagnetic constant for protons (γ_{I} =$2.675\times10^{+8}$ $\mathrm{T^{-1}s^{-1}}$); g is the electronic g-factor (g=2); S is the total electron spin of the Fe^{3+} ion (S= 5/2 for Fe^{3+}); μ_{B} is the Bohr magneton (μ_{B} = 9.274×10^{-24} $\mathrm{JT^{-1}}$); μ_{O} is the permeability of vacuum (μ_{O} =1.257×10^{-6} $\mathrm{NA^{-2}}$); r_{FeH} is the distance between the proton and the Fe^{3+} ; ω_{S} and ω_{I} are the angular electronic and proton Larmor frequencies (ω_{S} =658ω_{I} and ω_{I} =$\gamma_{\mathrm{I}}B$ where B is the magnetic field), A is the hyperfine coupling constant (in J) and η is the reduced Planck constant (η =$h/(2\pi)$=$1.054\times10^{-}$

34 Js).[3]

In equation SI-5, the two terms inside the square bracket (the "*3 term*" and the "*7 term*") have field dependence. The "*3 term*" is a function of the nuclear precession frequency while the "*7 term*" is a function of the electron precession frequency. Since the magnetogyric ratio is much larger for an electron than for a proton (γ_s/γ_H) =658, $\omega_s^2\tau_{c2}^2$ will become much greater than 1 at a much lower magnetic field than $\omega_I^2\tau_{c2}^2$. At the field where $\omega_s^2\tau_{c2}^2$ becomes greater than 1, the "7 term" disperses away to approach zero which has been proved in previous research.[3]

Additionally, because of the ionic nature of bonding in Fe^{3+} compounds (Fe^{3+}-OCPCs), and the fact that the water proton is separated from the Fe^{3+} ion by two bonds, the hyperfine coupling constant, *A*/*p*, is quite small. Thus the scalar mechanism in equation SI-5 is not very efficient; furthermore because of its $1/\omega_{s2}$ dependence it has dispersed at frequencies below 10 MHz. As a result, the characteristic time T_{1m} can be expressed through equation 9 shown in the main text.

The correlation times τ_{c1}, τ_{c2} and τ_e in equation SI-1 and SI-5 are defined as

$$\tau_{ci} = (\tau_R^{-1} + \tau_m^{-1} + T_{ie}^{-1})^{-1} \qquad \text{(SI-6)}$$

$$\tau_e = (\tau_m^{-1} + T_{2e}^{-1}); i = 1,2 \qquad \text{(SI-7)}$$

The time τ_R is the tumbling time of the complex and it can be estimated with the classical formula for spherical nanoparticles

$$\tau_R = \frac{4\mu\pi a^3}{3k_B T} \qquad \text{(SI-8)}$$

whereas the times T_{ie} are defined as

$$\frac{1}{T_{1e}} = \frac{1}{25}\Delta^2\tau_v[4S(S+1)-3]\left[\frac{1}{1+\omega_S^2\tau_v^2} + \frac{4}{1+4\omega_S^2\tau_v^2}\right] \qquad \text{(SI-9)}$$

$$\frac{1}{T_{2e}} = \frac{1}{25}\Delta^2\tau_v[4S(S+1)-3]\left[\frac{5}{1+\omega_S^2\tau_v^2} + \frac{2}{1+4\omega_S^2\tau_v^2} + 3\right] \qquad \text{(SI-10)}$$

where Δ^2 is the mean square zero field splitting (ZFS) energy and τ_v is the correlation time for splitting. The electronic ω_S and proton ω_I Larmor angular frequencies can be rephrased in the terms of the proton Larmor frequency ν_I as $\omega_I = 2\pi\nu_I$ and $\omega_S = 658\omega_I = 658(2\pi\nu_I)$. Clinically relevant magnetic field strength ranges between 0.25 and 3T, which corresponds to $\nu_I (= \gamma_I B/(2\pi))$ equal to about 10 and 130 MHz (for *B*=1.5T, $\nu_I \sim$ 65 MHz).[3]

The outer-sphere contribution to the longitudinal relaxivity r_1^{os} is given as

$$r_1^{OS} = \frac{1}{c_{Fe}}\left(\frac{1}{T_1}\right)_{OS} = \left(\frac{32\pi}{405}\right) C_{DD}\frac{N_A}{aD} Re[3j(\omega_I) + 7j(\omega_S)]$$

(SI-11)

where *Re* means real part and the complex function $j(\omega)$ is given by

$$j(\omega) = \left[4 + \left(i\omega\tau_\mathrm{D} + \frac{\tau_D}{T_{1e}}\right)^{1/2}\right] \Big/ \left[4 + 4\left(i\omega\tau_D + \frac{\tau_D}{T_{1e}}\right)^{1/2} + \frac{16}{9}\left(i\omega\tau_D + \frac{\tau_\mathrm{D}}{T_{1e}}\right) + \frac{4}{9}\left(i\omega\tau_D + \frac{\tau_\mathrm{D}}{T_{1e}}\right)^{3/2}\right] \quad \text{(SI-12)}$$

with the characteristic diffusion time τ_D defined as

$$\tau_\mathrm{D} = b_\mathrm{FeH}^2/D \quad \text{(SI-13)}$$

and *D* is the sum of the diffusion coefficients of bulk water and of Fe^{3+}-OCPCs; b_FeH is the distance of closest approach of the water molecules to Fe^{3+} in Fe^{3+}-OCPCs. It has recently been shown that the outer-sphere contribution can be neglected compared to the inner sphere contribution for sufficiently large fields (B>0.25T) and for slow tumbling construct which are generally associated with large inner-sphere relaxivities. Under these conditions the ratio r_1^{OS}/r_1^{IS} is generally smaller than 0.1. However, for paramagnetic complexes such as Fe^{3+}-OCPCs in our paper with small inner-sphere relaxivities, r_1^{OS}/r_1^{IS} could be close to unity.[3]

Discussion for the longitudinal relaxivity based on micromagnetic simulation and molecular rotational dynamics

The micromagnetic simulation and rotational dynamics analysis of paramagnetic Fe^{3+} ions were conducted to further reveal the mechanism of the T_1 contrast enhancement of Fe^{3+}-OCPCs. To simplify theoretical simulation and analysis process, we only considered the multilayered onion-like substructure of Fe^{3+}-OCPCs. As shown in **Figure S10**, compared to homogeneous Fe^{3+}-P2VP nanoparticles, the confinement of Fe^{3+} ions in the hierarchical microarchitecture of Fe^{3+}-OCPCs increases the local density of Fe^{3+} ions. Thus, the local density of Fe^{3+} in Fe^{3+}-OCPCs is larger than that in Fe^{3+}-P2VP. The increase in Fe^{3+} local density can shorten the distance between neighbor Fe^{3+} ions, further increasing magnetic dipolar interaction.

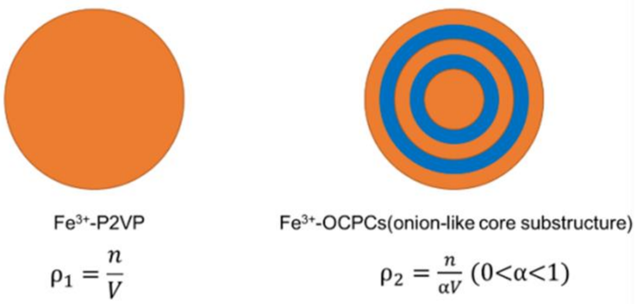

Figure S10. The schematic illustration of the density differences between Fe^{3+}-P2VP and the Fe^{3+}-OCPCs. The yellow part represents Fe^{3+}-P2VP domain while the blue part represents PEO domain. α represents the volume fraction of Fe^{3+}-P2VP layers in Fe^{3+}-OCPCs, which meets $0<\alpha<1$, making ρ_2 larger than ρ_1.

By modeling the Fe^{3+} ion as a paramagnetic sphere, we conducted the micromagnetic simulation and rotational dynamics analysis of paramagnetic Fe^{3+} ions. Here, we selected two extreme circumstances to simulate the influence of the average distance between neighbor Fe^{3+} ions. For the first circumstance where the distance between two paramagnetic Fe^{3+} ions was long enough (corresponding to Fe^{3+}-P2VP), we considered a 1×1 paramagnetic Fe^{3+} sphere arrangement, denoted as $(Fe^{3+})_i$. For the second circumstance where the distance between two Fe^{3+} ions was extremely short (corresponding to Fe^{3+}-OCPCs), we considered a 1×2 paramagnetic Fe^{3+} spheres arrangement, denoted as $(Fe^{3+})_i$ and $(Fe^{3+})_j$. The orientations of the overall magnetic spin structures of the paramagnetic Fe^{3+} spheres were visualized by using object oriented micromagnetic framework program (OOMMF, NIST). The principal calculation used in OOMMF simulation was based on the Landau-Lifshitz-Gilbert (LLG) equation.[4] The simulation results were obtained using the "Oxs_Uniform Exchange-Field" code. Based on the simulation results, we found that when two paramagnetic Fe^{3+} ions are approaching each other in a certain magnetic field, the non-deviated spins (white arrows) in the right edges of $(Fe^{3+})_i$ increased, indicating a stronger dipolar interaction between adjacent paramagnetic Fe^{3+} ions (**Figure S11**). Moreover, we also used the "Oxs_MinDriver-Spin" code to obtain more detailed and direct information of the spin structures of $(Fe^{3+})_i$ and $(Fe^{3+})_j$ by controlling minimization evolvers of internal spins (**Figure S12**). The black arrows in the middle of **Figure S12** become thicker, also indicating the stronger magnetic dipolar interaction.

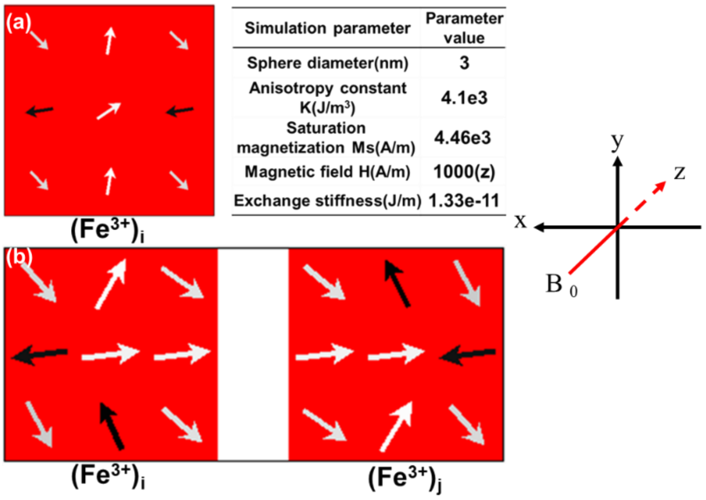

Simulation parameter	Parameter value
Sphere diameter(nm)	3
Anisotropy constant K(J/m³)	4.1e3
Saturation magnetization Ms(A/m)	4.46e3
Magnetic field H(A/m)	1000(z)
Exchange stiffness(J/m)	1.33e-11

Figure S11. (a-b) Simulated magnetic spin states of 1×1 Fe^{3+} paramagnetic sphere ($(Fe^{3+})_i$) arrangement and 1×2 Fe^{3+} paramagnetic spheres ($(Fe^{3+})_i$ and $(Fe^{3+})_j$) arrangement by using OOMMF program (Oxs Uniform Exchange-Field). The images were color-mapped according to the angle of the spin deviation versus the external magnetic field (which is parallel to the z axis), white indicated non-deviated spins and black indicates highly canted spins.

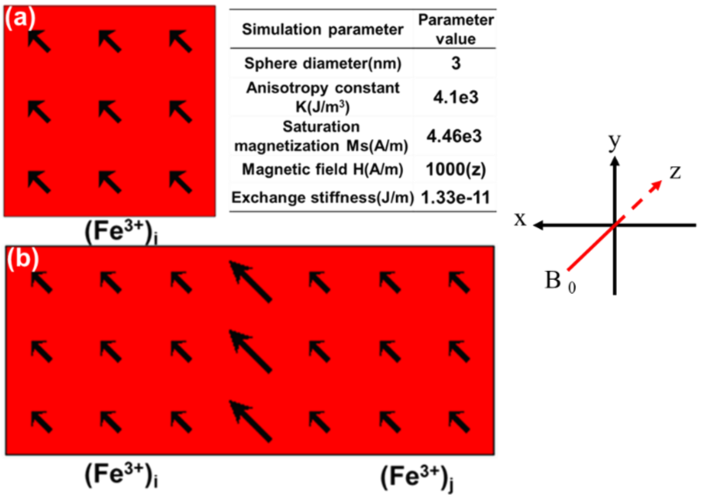

Figure S12. (a-b) Simulated magnetic spin states of 1×1 Fe^{3+} paramagnetic sphere ($(Fe^{3+})_i$) arrangement and 1×2 Fe^{3+} paramagnetic spheres ($(Fe^{3+})_i$ and $(Fe^{3+})_j$) arrangement by using OOMMF program (Oxs_MinDriver-Spin). The magnetic field is parallel to the z axis

Both "Oxs Uniform Exchange-Field code" and "Oxs_MinDriver-Spin" results indicate that the magnetic dipolar interaction is increased by shortening the distance between neighbor paramagnetic Fe^{3+} ions. Notably, the increased interaction between paramagnetic Fe^{3+} ions leads to a higher local viscosity around a Fe^{3+} ion.[5] Consequently, compared to homogeneous Fe^{3+}-P2VP nanoparticles, the confinement of Fe^{3+} ions in the hierarchical microarchitecture of Fe^{3+}-OCPCs increases the magnetic dipolar interaction and local viscosity of paramagnetic Fe^{3+} ions.

The T_1 relaxation times of water protons induced by a single Fe^{3+} electronic spin can be described as:[6]

$$\frac{1}{T_1} = \frac{1}{T_{1,bulk}} + \sum_i 3\,\gamma_i^2 B_{\perp,i}^2 \frac{\tau_{c,i}}{1+\omega_0^2\tau_{c,i}^2} \quad \text{(SI-1)}$$

$$\tau_{c,i} = 1/R_i \text{ (SI-2)}$$

where $T_{1,\,bulk}$ is the relaxation time of Fe^{3+} in Fe^{3+}-OCPCs and ω_0 is the energy level splitting between the $|m_s = 0\rangle$ and $|m_s = \pm 1\rangle$ states at zero external magnetic field. For each

paramagnetic Fe^{3+} ion *i*, γ_i is the gyromagnetic ratio; $B^2_{\perp,i}$ is the rms transverse magnetic noise strength induced by neighbor Fe^{3+}; and $\tau_{c,i} = 1/R_i$ is the noise correlation time of neighbor Fe^{3+}, the inverse of the noise fluctuation rate (R_i) [7] Since the rotational motion rates of paramagnetic Fe^{3+} ions are typically in GHz range, the value of R_i would be larger than ω_0 When the fluctuation rate of the neighbor paramagnetic Fe^{3+} paramagnetic ion is larger than the resonant frequency of the Fe^{3+} spin ($R_i > \omega_0$), a further increase in fluctuation rate results in a smaller noise spectrum intensity at the spin resonance frequency, further leading to a longer T_1 relaxation time of the water protons. The higher local viscosity induced by increasing dipolar interaction between neighbor paramagnetic Fe^{3+} ions can decrease R_i, further increasing r_1 relaxivity. Consequently, the increase in the local viscosity for Fe^{3+}-OCPCs lead to the enhancement of magnetic dipolar interaction between neighbor paramagnetic Fe^{3+} ions, thus resulting in the decreased R_i value and the improved T_1 contrast effect.

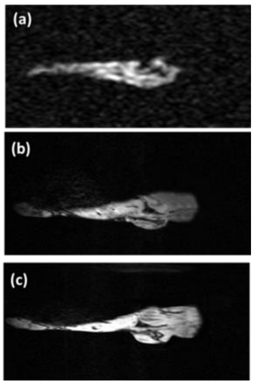

Figure S13. High-spatial-resolution MR T1-weighted contrast image of Fe^{3+}-OCPCs-injected zebrafish embryo (98 hours after fertilization) obtained at 7 T. (a) Gd(DTPA), (b) $Fe^{3+}_{0.06}$-P2VP and (c)$Fe_{0.06}^{3+}$-OCPCs.

The Demonstration of the novelty of the concept "nano-metamaterials"

nanomaterials have been widely applied in biological field. Nevertheless, there are no reports on the application of artificially structured nano-metamaterials with multilevel ordered microarchitectures in biomedical field. We searched on Web of Science Core Collection with key word 'nano-metamaterials' for related publications. Only 18 reports (except for 3 reviews and 1 journal article) popped up using the concept of nano-metamaterials (https://www.webofscience.com/wos/alldb/summary/591c5407-baef-4508-a626-96085f5444f0-3047f97a/relevance/1). In this field, the "ferroelectric nano-metamaterials" was

an important research topic, accounting for almost 39% of the publications. For example, the concept of ferroelectric nano-metamaterials was firstly proposed and published on Scientific Reports.[11] The concept of nano-metamaterials was further applied in sensing such as Terahertz sensing[12] or shielding,[13] with a publication share of 33%. Other optical applications were also involved, such as photonic devices[14] and solar absorber.[15] These results indicated that the nano-metamaterials, as a branch concept of metamaterials, have not gained strong attention and were mainly applied for physics applications. Despite the concept of nano-metamaterials has been mentioned in different researches concerning ferroelectric materials and optical materials, we summarized that the researches in the past decades have three characteristics: (i) Numerical studies and computational analyses have been the mainstream while the synthesis protocols have not been widely discussed; (ii) Those nano-metamaterials were always a two-dimensional (2D) nano-specimens consisting of numerous ordered building blocks, whereas the well-defined definition of nano-metamaterials has not been summarized and given, especially considering the microstructure of nano-metamaterials would further evolve from 2D microarchitecture to 3D microarchitecture; (iii) The applications of nano-metamaterials evolved from ferroelectric to the optical fields < Int. J. Mech. Sci., 2020, 184 >, low regard was held in the biological field. The experimental design, synthetic methods or the microarchitecture-biological performance relationship of nano-metamaterials for biological application have not been discussed. These results indicated that the nano-metamaterials, as a branch concept of metamaterials, have not gained strong attention and were mainly applied for physics applications. Therefore, an accessible design principle for 3D nano-metamaterials, which would further extend their application towards biology, is important for the development of nano-metamaterials and will provide inspirations for technicians and scientists to design novel classes of nano-metamaterials. On the basis of those considerations, we draw on a novel dual-kinetic controlled strategy to synthesize 3D nano-metamaterials, which comprised three parts: (i) the homogeneous interior core; (ii) an onion-like shell; and (iii) a hierarchically porous corona. We defined that nano-metamaterials were rationally designed materials with multilevel multiscale microarchitectures and both characteristic sizes and whole sizes at nanoscale, investing in themselves remarkably unique and significantly enhanced material properties, such as optical, ferroelectric and biological properties as compared with conventional nanoparticles. Compared to these previous reported works, our manuscript proposed a clear definition and novel synthesis protocol for the nano-metamaterials with 3D microarchitecture. Furthermore, we extended the application of nano-metamaterials to biological fields, manifesting a microarchitecture-biological performance

relationship of nano-metamaterials. It should be noted that the remarkable properties were originated from the hierarchical complexity, as well as the intrinsic properties of the constituent chemicals.

Table S1. Molecular characteristics of the Amphiphilic Diblock Copolymers

Block Copolymer	M_n	PDI	PEO content%
PEO-*b*-P2VP	13000	1.13	77

Table S2. Polymer/Solvent Solubility Parameter[16-18]

Chemical	Solubility parameter[MPa]$^{1/2}$
PEO	21.6
P2VP	22.4
Dichloromethane	19.8
H_2O	47.3

The suitable cosolvent (dichloromethane) of PEO-*b*-P2VP was found through theoretical analysis. The Flory–Huggins model is expressed interaction parameters for polymer–solvent and polymer–polymer segments at temperature T, which can be expressed as follows:

$$\chi_{polymer-solvent} = V_{solvent}\left(\delta_{polymer} - \delta_{solvent}\right)^2/RT + 0.34$$

where the $\chi_{polymer-solvent}$ symbol represents polymer–solvent interaction parameter at absolute temperature T, $V_{solvent}$ represents molar volume of solvent, R is the gas constant, δ present solubility parameters of solvent and polymer (Table S2).[19] According to Flory Huggins theory standard, when $\chi_{polymer-solvent} < 0.5$, the polymer completely dissolved in the solvent among the whole composition range.

References

[1] A. S. Utada, E. Lorenceau, D. R. Link, P. D. Kaplan, H. A. Stone, D. A. Weitz, *Science* **2005**, *308*, 537.

[2] J. S. Ananta, B. Godin, R. Sethi, L. Moriggi, X. W. Liu, R. E. Serda, R. Krishnamurthy, R. Muthupillai, R. D. Bolskar, L. Helm, M. Ferrari, L. J. Wilson, P. Decuzzi, *Nat. Nanotechnol.* **2010**, *5*, 815.

[3] P. Caravan, J. J. Ellison, T. J. McMurry, R. B. Lauffer, *Chem. Rev.* **1999**, *99*, 2293.

[4] U. Bajpai, B. K. Nikolić, *Phys. Rev. B* **2019**, *99*, 134409.

[5] X. N. Ma, L. Y. Yan, X. F. Wang, Q. J. Guo, A. D. Xia, *J. Phys. Chem. A* **2011**, *115*, 7937.

[6] J. P. Tetienne, T. Hingant, L. Rondin, A. Cavailles, L. Mayer, G. Dantelle, T. Gacoin, J. Wrachtrup, J. F. Roch, V. Jacques, *Phys. Rev. B* **2013**, *87*, 235436.

[7] S. Okada, B. B. Bartelle, N. Li, V. Breton-Provencher, J. J. Lee, E. Rodriguez, J. Melican, M. Sur, A. Jasanoff, *Nat. Nanotechnol.* **2018**, *13*, 473.

[8] S. Steinert, F. Ziem, L. T. Hall, A. Zappe, M. Schweikert, N. Gotz, A. Aird, G. Balasubramanian, L. Hollenberg, J. Wrachtrup, *Nat. Commun.* **2013**, *4*, 1607.

[9] J. H. Van Vleck, *Phys. Rev.* **1948**, *74*, 1168.

[10] S. Rast, P. H. Fries, E. Belorizky, *J. Chem. Phys.* **2000**, *113*, 8724.

[11] T. Shimada, L. Van Lich, K. Nagano, J. Wang, T. Kitamura, *Sci. Rep.* **2015**, *5*, 14653.

[12] D. K. Lee, J. H. Kang, J. Kwon, J. S. Lee, S. Lee, D. H. Woo, J. H. Kim, C. S. Song, Q. H. Park, M. Seo, *Sci. Rep.* **2017**, *7*, 8146.

[13] G. Choi, F. Shahzad, Y. M. Bahk, Y. M. Jhon, H. Park, M. Alhabeb, B. Anasori, D. S. Kim, C. M. Koo, Y. Gogotsi, M. Seo, *Adv. Opt. Mater.* **2018**, *6*, 1701076.

[14] Q. Wang, L. Yu, H. X. Gao, S. W. Chu, W. Peng, *Opt. Express* **2019**, *27*, 35012.

[15] J. F. Liu, J. Chen, H. Liu, Y. Y. Liu, L. Zhu, *Plasmonics* **2020**, *15*, 1517.

[16] A. Forster, J. Hempenstall, I. Tucker, T. Rades, *Int. J. Pharm.* **2001**, *226*, 147.

[17] D. R. Lide, CRC handbook of chemistry and physics. 77th ed. New York: CRC Press; 1996 p. 6-151.

[18] J. Brandrup, E. H. Immergut, 3rd ed Polymer handbook. vol. VII. New York: Wiley-Interscience; 1989 p. 526.

[19] H. B. Du, J. T. Zhu, W. Jiang, *J. Phys. Chem. B* **2007**, *111*, 1938.
